# Evolutionary refinement of the ZIKV 5′ UTR dictates a trade-off between RNA stability and promoter accessibility

**DOI:** 10.1093/narmme/ugag027

**Published:** 2026-05-22

**Authors:** Alex B Wang, Quinn H Abram, Carolina Camargo, Trisha R Barnard, Selena M Sagan

**Affiliations:** Department of Microbiology & Immunology, University of British Columbia, Vancouver, BC V6T 1Z3, Canada; Department of Biochemistry, McGill University, Montréal, QC H3A 1A3, Canada; Department of Microbiology & Immunology, University of British Columbia, Vancouver, BC V6T 1Z3, Canada; Department of Biochemistry, McGill University, Montréal, QC H3A 1A3, Canada; Department of Microbiology & Immunology, University of British Columbia, Vancouver, BC V6T 1Z3, Canada; Department of Medicine, Stanford University School of Medicine, Stanford, CA 94305-5107, United States; Department of Microbiology & Immunology, McGill University, Montréal, QC H3A 1A3, Canada; Department of Microbiology & Immunology, University of British Columbia, Vancouver, BC V6T 1Z3, Canada; Department of Biochemistry, McGill University, Montréal, QC H3A 1A3, Canada; Department of Microbiology & Immunology, McGill University, Montréal, QC H3A 1A3, Canada

## Abstract

Zika virus (ZIKV) has evolved from a sporadically circulating pathogen into a global health threat associated with severe neurological complications. While research has been focused on amino acid substitutions, the impact of non-coding RNA evolution on ZIKV fitness remains largely unexplored. Here, we characterize the structural and functional evolution of stem-loop A (SLA), the essential 5′ terminal promoter for the viral polymerase. We identified four distinct evolutionary variants, ancestral (Anc), intermediate (Int), contemporary (Con), and alternative (Alt) that exhibit a progressive shift in thermodynamic stability. Notably, SLA^Con^—dominant in recent outbreaks—displays increased binding affinity for the NS5 polymerase, whereas the divergent SLA^Alt^ variant markedly increases 5′ exoribonuclease resistance at the cost of replicative efficiency. Although the SLA^Alt^ variant proved lethal in a contemporary ZIKV backbone, 5′ rapid amplification of cDNA ends (RACE) and phylogenetic analysis confirmed its viability in nature. These findings reveal a previously unrecognized plasticity in the ZIKV promoter and demonstrate that non-coding architecture undergoes adaptive refinement to balance genome stability with kinetic accessibility. Ultimately, this work illustrates how the structural evolution of RNA serves as a key determinant of viral fitness and the emergence of isolate-specific replication strategies.

## Graphical Abstract

**Graphical Abstract:**
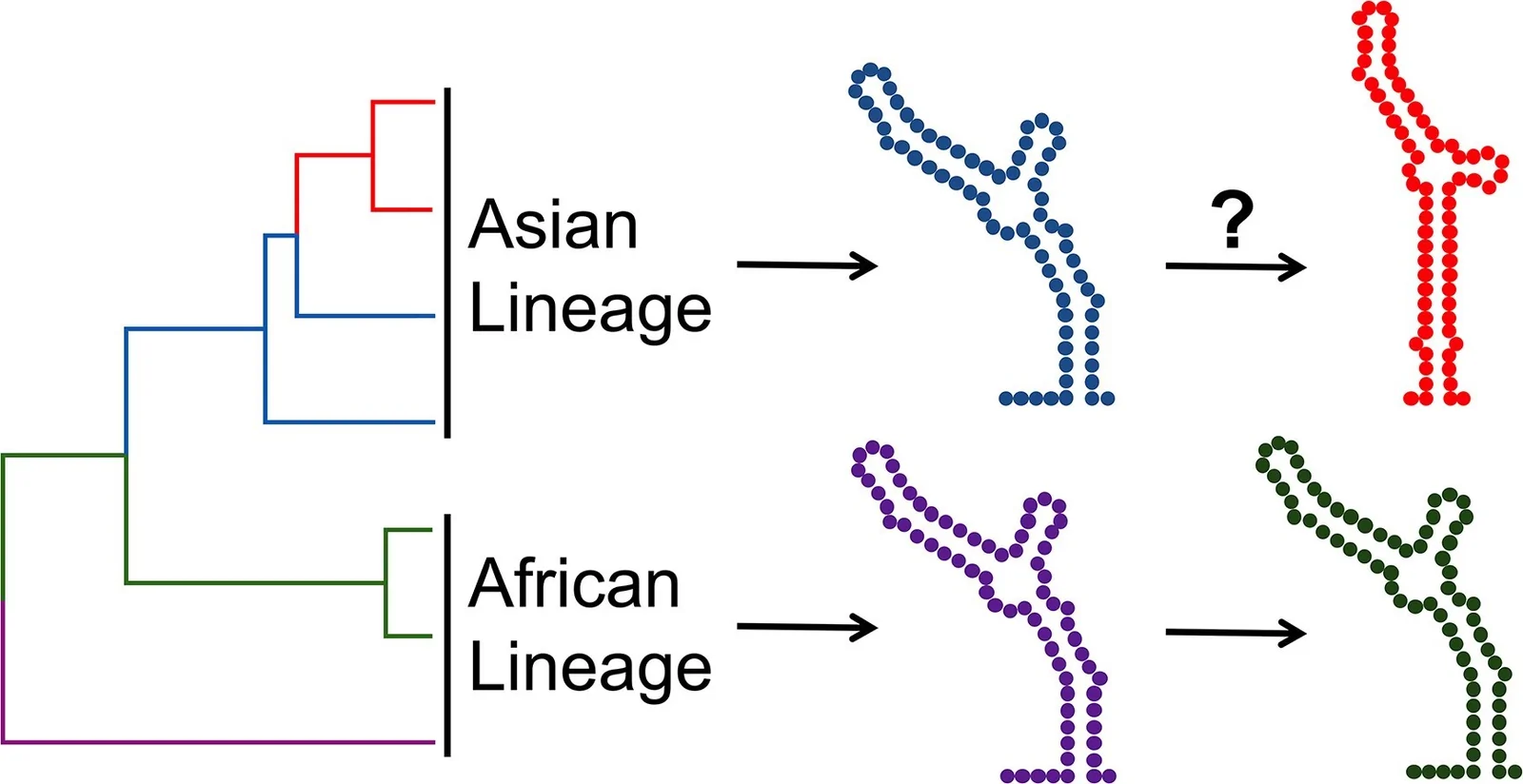

## Introduction

Zika virus (ZIKV), a mosquito-borne positive-sense RNA virus, transitioned at the beginning of the 21st century from decades of sporadic circulation to a global public health priority associated with severe neurological complications, including Guillain–Barré syndrome and fetal microcephaly [1–4]. This expansion coincided with the divergence of ZIKV into two distinct lineages: the African and Asian lineages [5, 6]. While investigations into ZIKV pathogenesis have largely focused on amino acid polymorphisms, the contribution of non-coding RNA evolution to viral fitness and genome regulation remains unclear [7, 8].

The orthoflavivirus 5′ and 3′ untranslated regions (UTRs) are sophisticated regulatory hubs that coordinate translation, replication, and genome stability [9–12]. At the 5′ terminus, stem-loop A (SLA) serves as the essential promoter for the viral RNA-dependent RNA polymerase (RdRp), NS5 [9]. Successful initiation of viral RNA synthesis requires NS5 recruitment to SLA, with subsequent cyclization of the viral RNA contributing to ribosome exclusion from the viral genome [13–15]. Additionally, this genome cyclization helps position the NS5 polymerase at the 3′ end of the genome for the initiation of negative-strand RNA synthesis [9, 16, 17]. Beyond its role in viral RNA synthesis, the SLA architecture is also critical for genome capping, where it facilitates the precise orientation of the 5′ terminus for N-7 and 2′-*O*-methylation by NS5’s methyltransferase activity [9, 18, 19].

Despite the functional necessity of the 5′ UTR, the structural plasticity of SLA across naturally occurring ZIKV isolates is poorly defined. Most models assume a static, “Y”-shaped SLA architecture; however, the impact of lineage-specific nucleotide polymorphisms on the mechanical flexibility or thermodynamic stability of this promoter is unknown. Given that the 3′ UTR is already recognized as a site of functional evolution—producing subgenomic flaviviral RNAs (sfRNAs) that modulate host immunity—it is likely that the 5′ UTR similarly undergoes adaptive structural refinement [7, 20–24].

In this study, we analyzed ZIKV genomic evolution and identified four distinct SLA variants: ancestral (SLA^Anc^), intermediate (SLA^Int^), contemporary (SLA^Con^), and a divergent alternative (SLA^Alt^) form. Using a combination of *in silico* modeling, *in vitro* structural probing, and reverse genetics analyses, we demonstrate that these variants exhibit a progressive shift in RNA stability along the evolutionary trajectory. Moreover, we identified a divergent variant, SLA^Alt^ that displays a distinct conformation and significantly increased exoribonuclease resistance, yet proved to be replication-defective in a contemporary backbone, suggesting a high degree of context dependency in ZIKV promoter function. Nonetheless, we were able to identify a replication-competent clinical isolate harbouring the SLA^Alt^ variant. Collectively, these findings highlight the functional and evolutionary significance of structural diversity within the ZIKV 5′ UTR. The identification of a naturally occurring SLA^Alt^ variant that is replication-defective in contemporary systems suggests that the non-coding architecture is significantly more plastic than previously recognized. Such structural flexibility likely serves as a key determinant of isolate-specific replication kinetics and underscores the complex adaptive landscape of the orthoflavivirus genome.

## Materials and methods

### Sequence collection and datasets

Four hundred and eighty sequences of natural ZIKV isolates >10 600 nucleotides (nts) in length collected from primates or mosquitos between 1 January 1947 and 2 February 2024 deposited on NCBI’s GenBank database (https://www.ncbi.nlm.nih.gov/GenBank/) were compiled and aligned to the ZIKV/*H. sapiens*/Brazil/Natal/2015 (GenBank Accession: NC_035 889) reference sequence in MAFFT ver. 7 (https://mafft.cbrc.jp/alignment/server/) using the L-INS-i algorithm to generate *Dataset 1*. Identically named isolates were trimmed to one representative isolate. To generate *Dataset 2, Dataset 1* was further refined to retain only the 97 sequences containing the entire SLA sequence (nts 4–70 of the ZIKV genome). As an additional subset of *Dataset 1, Dataset 3* contained only those sequences beginning at or before nucleotide 8 of the ZIKV genome; sequences missing >5% total nucleotide identities or missing >50 consecutive nucleotides were removed, resulting in 105 sequences.

### Nucleotide variability analyses

Using *Dataset 2*, the percent deviation from the consensus sequence at each nucleotide was calculated. Using the same sequences, sequence logos were generated using WebLogo (https://weblogo.berkeley.edu/logo.cgi).

### Phylogenetic analyses

Amino acid sequences were compiled using amino acid accession numbers from *Dataset 3*. These sequences were then aligned using MUSCLE and used to construct a maximum-likelihood tree using MEGA11 [25, 26]. Bootstrap resampling was used to determine robustness of branches (500 replicates). Proportions of each SLA variant as well as their proportions with respect to each lineage were calculated using *Dataset 3* and graphed using the GraphPad Prism 10 software (GraphPad, San Diego, CA, USA).

### RNA secondary and tertiary structural predictions

RNA structural predictions were performed on the 5′ UTR (nts 1–107) of ZIKV sequences. Predictions for SLA (nts 1–70) are shown. ZIKV strain MR766 (GenBank accession: KX830960), Zika virus/A.africanus-tc/SEN/1984/41525-DAK (GenBank accession: KU955591), PRVABC-59, (GenBank accession: KX377337), and Haiti/1225/2014 (GenBank accession: KU509998), were used as representative strains for SLA^Anc^, SLA^Int^, SLA^Con^, and SLA^Alt^, respectively. To obtain the predicted Gibbs free energy (Δ*G*) of each SLA variant, nts 1–70 were used.

RNA secondary structure and Gibbs free energy predictions were performed using the RNAstructure software package from the Mathews Lab, available at https://rna.urmc.rochester.edu/index.html [27]. ZIKV genomic 5′ UTR RNA sequences of interest were loaded into the RNAstructure software using the “Fold RNA Single Strand” command. The output was saved as a dot bracket file and was visualized using VARNA [28].

RNA tertiary structure predictions of SLA (nts 1–70) of the ZIKV genome of the different SLA variants of interest were performed using AlphaFold 3 Server (https://alphafoldserver.com/ [29]). The five predicted structures with the highest AlphaFold ranking score were aligned and visualized in PyMol [30].

### Biological resources

African green monkey kidney (Vero) cells and human lung carcinoma (A549) cells were kindly provided by Martin J. Richer (University of Indiana, IN, USA) and Russell Jones (Van Andel Institute, MI, USA), respectively. All cells were maintained in Dulbecco’s Modified Eagle’s Medium (DMEM) supplemented with 10% fetal bovine serum (FBS), 1% nonessential amino acids, and 1% L-glutamine (DMEM Complete Media). Huh-7.5 cells were maintained similarly, as previously described [31]. All cells were maintained at 37°C/5% CO_2_ and were routinely screened for mycoplasma contamination.

An infectious cDNA of ZIKV strain PRVABC59 (ZIKV^PR^; GenBank accession: KX377337) was kindly provided by Young-Min Lee (Utah State University, UT, USA [32]). The ZIKV isolates, including PRVABC59 (ZIKV^PR^, GenBank accession: KU501215) and HS-2015-BA-01 (ZIKV^BR^, GenBank accession: KX520666) were provided by Tom Hobman (University of Alberta, AB, Canada) and Mauro Teixeira (Universidade Federal de Minas Gerais, MG, Brazil), respectively.

### Plasmids and cloning

The ZIKV 5′ UTR plasmid for producing *in vitro* transcribed (IVT) RNA for secondary structural analyses contains the first 383 nts of the ZIKV PRVABC59 genome flanked by a T7 promoter (T7; 5′-TAA TAC GAC TCA CTA TAG-3′) and the hepatitis delta virus ribozyme (HDVr; 5′-GGG TCG GCA TGG CAT CTC CAC CTC CTC GCG GTC CGA CCT GGG CTA CTT CGG TAG GCT AAG GGA GAA G-3′). The T7 through HDVr sequence was produced as a gBlock (IDT) and inserted into the pUC18 backbone using EcoRI and BamHI. Subsequently, the first 383 nts of the ZIKV PRVABC59 genome was inserted via Gibson assembly using the following primer pairs: ZIKV 5(+) GA Insert Fwd and ZIKV 5(+) GA Insert Rev, as well as pUC18 SVP RPCR Fwd and (+)163nt Vector RPCR Rev (Supplementary Table S1). Mutations in this region corresponding to the SLA^Anc^ (A19G, T41C, A50G, G56A; SLA-G19A-C41U-RPCR-Fwd + SLA-G19A-C41U-RPCR-Rev and SLA-G50A-A56G-RPCR-Fwd + SLA-G50A-A56G-RPCR-Rev), SLA^Int^ (A50G, G56A; SLA-G50A-A56G-RPCR-Rev and SLA-G50A-A56G-RPCR-Rev), and SLA^Alt^ (G8A, A9C, C11G, G13T, T14G, G15C, A19C; pos363IVT-SLAalt-RPCR-For and pos363IVT-SLAalt-RPCR-Rev) sequences were introduced via reverse PCR with Q5 Hot Start High-Fidelity 2X Master Mix [New England Biolabs (NEB), Supplementary Table S1]. The resulting PCR products were treated with KLD Enzyme Mix (NEB). The Gibson assembly reaction was then transformed into NEB 5-alpha Competent *Escherichia coli* (NEB), and colonies were screened. Plasmids were purified with the QIAprep Spin Miniprep kit (Qiagen) following manufacturer’s instructions and verified by Sanger sequencing (Sequencing + Bioinformatics Consortium).

The ZIKV PRVABC59 subgenomic replicon consists of the ZIKV 5′ UTR and the first 38 amino acids of capsid followed by a *Renilla* Luciferase (RLuc) reporter gene, the Foot and Mouth Disease Virus (FMDV) 2A peptide, the last 30 amino acids of Envelope, and the remainder of the ZIKV genome from NS1 to the 3′ UTR [33]. To generate each SLA mutant in the ZIKV PRVABC59 subgenomic replicon, the region from the PacI to ApaLI restriction sites were subcloned into a pUC18 vector. Each mutation was introduced into the subcloned plasmid by reverse PCR, as described above. The SLA^Anc^ and SLA^Int^ mutations were introduced using primers described above. The SLA^Alt^ mutations were introduced using SLA-Alt-RPCR-For-1 and SLA-Alt-RPCR-Rev-1 (Supplementary Table S1). The subcloned plasmid was then digested with PacI and ApaLI and the fragment was ligated into the WT or GNN ZIKV PRVABC59 subgenomic replicon using T4 DNA Ligase (NEB) according to manufacturer’s instructions. The resulting SLA variant ZIKV subgenomic replicon bacterial artificial chromosome was purified using the QIAGEN Large-Construct kit (Qiagen). Mutations were confirmed by Oxford Nanopore sequencing (Plasmidsaurus).

To generate the IVT template for *in vitro* exoribonuclease assays, the T7 promoter of the ZIKV 5′ UTR plasmid was mutated to a class II T7 promoter (T7II; 5′- TAA TAC GAC TCA CTA TTA-3′) via reverse PCR, as described above, to allow for transcription initiation on an Adenine. Forward primer ZIKV-WT-IVT-T7II-For and reverse primer ZIKV-puc18-IVT-T7II-Rev were used for SLA^Anc^, SLA^Int^, and SLA^Con^, while the forward primer ZIKV-Alt-IVT-T7II-For was used for the SLA^Alt^-encoding plasmid (Supplementary Table S1).

### *In vitro* transcription

To generate IVT RNA for *in vitro* selective 2′ hydroxyl acylation analyzed by primer extension (SHAPE) analysis, the 480 nt DNA template was generated via PCR using primers EcoRI-T7-For (5′- GGC CAG TGA ATT CTA ATA CGA CTC ACT ATA -3′) and BamHI-HdvR-Rev (5′- TAT GGA TCC CTT CTC CCT TAG CCT ACC GAA G -3′). The resulting product was agarose gel extracted using the QIAquick Gel Extraction Kit (Qiagen). IVTs were performed using the T7 Ribomax Express Large Scale RNA Production System (Promega) according to manufacturer’s instructions. Full-length IVT RNAs were separated on a 6% Urea-PAGE gel at 4ºC and eluted overnight at 4ºC with gentle agitation in 400 µl elution buffer [500 millimolar (mM) NH_4_OAc, 10 mM EDTA and 0.1% sodium dodecyl sulphate (SDS)]. Eluted RNA was ethanol precipitated in 2.5 volumes of 95% ethanol and 0.1 volumes 3 molar (M) sodium acetate, pH 5.2 and stored at −70ºC until use. The same process was followed to generate the corresponding IVT RNA using templates containing the SLA^Anc^, SLA^Int^, and SLA^Alt^ mutations in the context of the PRVABC59 genomic sequence.

To generate ZIKV subgenomic replicon RNAs, all templates were linearized with PsrI (Creative Enzymes), verified by agarose gel electrophoresis, and column-cleaned using the Zymo DNA Clean and Concentrator kit (Zymogen) according to the manufacturer’s instructions. Two hundred and fifty nanograms (ng) of linearized template DNA was incubated in a 60 µl reaction at 37°C for 2 h with 60 units (U) SP6 polymerase (NEB), 1× RNAPol reaction buffer, 60 U RiboLock (Invitrogen), 5 mM DTT, 1 mM each ATP, UTP, CTP, and GTP, as well as 0.8 mM m^7^GpppA cap analog (NEB). IVT RNA was DNase I (NEB) treated, verified by agarose gel electrophoresis, and column-cleaned using the Zymo RNA Clean & Concentrator kit (Zymogen). RNA was stored at −70°C until use.

To generate capped Firefly luciferase (FLuc) mRNA (control), the pT7Luc plasmid (Promega) was linearized with XmnI and IVT using the mMESSAGE mMACHINE T7 Transcription Kit (Invitrogen) according to manufacturer’s instructions. RNA was precipitated and stored as described above. All nucleic acid concentrations were determined by UV–Vis spectrophotometry at 260 nm (Nanodrop).

To generate IVT RNA for the *in vitro* exoribonuclease assay, the 93 nt DNA template was generated via PCR using primers EcoRI-T7II-For (5′- GAA TTC TAA TAC GAC TCA CTA TTA -3′) and ZIKV-+IVT-70nt-Rev (5′- TGT TGA TAC TGT TGC TAG CTT TCG -3′). The resulting product was agarose gel extracted using the QIAquick Gel Extraction Kit (Qiagen). Three hundred nanograms of PCR product was incubated in a 50 µl reaction at 37°C for 2 h with 50 U T7 polymerase (NEB), 1× RNAPol reaction buffer, 50 U RiboLock (Invitrogen), 1 mM each UTP, CTP, and GTP, as well as 1.2 mM ATP (NEB). IVT RNA was DNase I (NEB) treated, verified by agarose gel electrophoresis, and ethanol precipitated as above. RNA was stored at −70°C until use.

### *In vitro* shape

*In vitro* SHAPE was performed in experimental quadruplicate as previously described, with the following modifications [34]. In brief, 5 picomole (pmol) of ZIKV RNA (nts 1–383) was re-folded and incubated in SHAPE buffer (333 mM HEPES, pH 8.0; 20 mM MgCl_2_; 333 mM NaCl). RNA was then exposed to 0.01 M NAI-N_3_ or dimethyl sulfoxide (DMSO, treatment control) for 5 min at 37ºC, and then extracted using TriZol reagent (ThermoFisher Scientific) according to the manufacturer’s instructions. Extracted RNA was precipitated with 2.5 volumes 95% ethanol, 0.1 volumes 3 M sodium acetate, pH 5.2, and stored at −70ºC. RNA was used for SHAPE analysis by capillary electrophoresis, as previously described, with the following modifications [34]. Primer extensions were performed in a total reaction volume of 20 µl using 5 pmol NAI-N_3_-labelled ZIKV RNA and 1 pmol of the 6-FAM-labeled ZIKV (+) 5UTR PE (5′- CAG CAT GGC AGC CAG ATC TTT CTT -3′). Matched sequencing ladders were generated by performing primer extension reactions using 5 pmol unlabelled ZIKV RNA, 1 pmol of the NED-labeled ZIKV (+) 5UTR PE oligo, and either 0.5 mM ddCTP or ddGTP. Following the 30 min primer extension at 52°C, 2 M NaOH was added and incubated at 95°C for 5 min to degrade input RNA. The remaining cDNA was then diluted to a total volume of 100 µl with water and ethanol precipitated as above. Pelleted cDNA was washed three times with cold 85% ethanol and dried at 37°C for 10 min. Dried pellets were sent for capillary electrophoresis on an ABI 3100 Genetic Analyzer at Plateforme d’Analyses Génomiques de l’Université Laval. Raw fluorescence data was analysed using the QuSHAPE software package [35]. The resulting normalized SHAPE data was used as a constraint to generate RNA secondary structure predictions using the RNAstructure software package [27]. RNA sequences were loaded as a “Single Sequence” using the “Structure Prediction” command, with the normalized SHAPE data then input as a constraint on secondary structure prediction as a “.shape” file. Outputs were saved as dot bracket files and visualized via VARNA.

### Electrophoretic mobility shift assay

ZIKV NS5 EMSAs were performed as previously described [36]. In brief, 2.5 pmol (150 ng) of IVT RNA corresponding to the 163 nt at the 5′ terminus of the positive-strand that includes SLA was heated at 95ºC and then snap cooled on ice. Once cooled, RNA was added to a binding reaction with a total reaction volume of 20 μl containing one dilution of a series of ZIKV NS5 ranging from a molar ratio (NS5:RNA) of 0–8:1 (0–2 μg of NS5) in NS5 binding buffer. Binding reactions proceeded at 25ºC for 30 min before separation on a 1% agarose 0.5× TBE gel run for 1 h at 100 V on ice at 4ºC. Gels were stained with 1× SYBR Gold nucleic acid gel stain (Thermo Fisher) on an orbital shaker at 120 rpm for 20 min and visualized on a ChemiDoc MP Imaging System (Bio-Rad).

### Electroporation

A549 cells were trypsinized and washed twice with cold phosphate-buffered saline (PBS, Gibco). Four million cells were resuspended in 800 µl of cold PBS, mixed with 5 µg capped ZIKV subgenomic replicon IVT RNA, and 1 µg capped and polyadenylated FLuc mRNA (electroporation control) in a cold 4-mm electroporation cuvette [31, 34]. Cells were electroporated with a single square wave pulse at 250 V for 20 ms, optimized for the Bio-Rad Gene Pulser XCell (Bio-Rad). After a 10 min recovery at room temperature, the electroporated cells were resuspended in DMEM complete media and 2 ml per timepoint were plated in either 15 ml Falcon tubes (for 2–8 h timepoints), 12-well (24 h timepoint) or 6-well plates (48–72 h timepoints). Where indicated, RLuc signals were normalized to the 2 h FLuc signal to account for electroporation efficiency.

### Luciferase assay

Cells were washed with cold PBS and harvested in 100 µl of 1× Passive Lysis Buffer (Promega). The Dual Luciferase Assay Reporter Kit (Promega) was used to analyse luciferase activity according to the manufacturer’s protocol, with the following modifications. Luciferase assays were performed in opaque, white 96-well plates using 10 µl of sample per well and 50 µl of luciferase reagents. A GloMax Explorer plate reader (Promega) was used to measure luminescence with a 10 s integration time. Each sample was read in technical duplicate. Firefly luciferase was used to ensure similar electroporation efficiency across samples.

### *In vitro* exoribonuclease assays

A total of 12 µg triphosphorylated 70 nt IVT RNA of each SLA variant were 5′ Pyrophosphohydrolase (RppH; NEB) treated for 1 h according to manufacturer’s instructions to generate monophosphorylated SLA RNAs. Monophosphorylated RNAs were then column-cleaned using the Zymo RNA Clean & Concentrator kit and refolded by denaturing at 65°C for 3 min followed by incubation at 37°C for 10 min. Xrn-1 assays were performed with 1 U Terminator 5′ Phosphate-Dependent Exonuclease in 1× Terminator Reaction Buffer A (Biosearch Technologies) and 20 U Ribolock. Reactions were incubated at 30°C and quenched with 1 µl of 100 mM EDTA at the indicated timepoints. Subsequently, 1× RNA Loading Dye (NEB) was added to each sample, and samples were resolved on 10% denaturing urea polyacrylamide gels. Gels were visualized by SYBR Gold (ThermoFisher) staining and imaged on a ChemiDoc MP Imaging System (Bio-Rad). Three experimental replicates were performed, and representative gels are shown.

### Virus infections

Propagation and titering of the Asian lineage isolates (ZIKV^PR^ and ZIKV^BR^) have been previously described [38]. For infections, human hepatoma (Huh-7.5) cells were seeded at a density of 2.5 × 10^6^ cells per plate in 10 cm plates the day before infection. Viral infections were performed at multiplicity of infection (MOI) of 3 in EMEM (ATCC) and incubated on subconfluent monolayers of cells at 37°C/5% CO_2_. At 1 h post-infection, the inoculum was removed, cells were washed once with PBS, and the media was replaced with fresh complete media containing 15 mM HEPES (Gibco) and 2% FBS. Cells were harvested in 1 ml of TriZol Reagent (ThermoFisher) 24 h post-infection according to manufacturer’s instructions.

### 5′ rapid amplification of cDNA ends (RACE) analysis

Total RNA was extracted from cells using TRIzol Reagent according to manufacturer’s instructions. Ten micrograms total RNA was treated with 10 U Antarctic Phosphatase (NEB) in the presence of 40 U RiboLock and incubated at 37°C for 1 h. Subsequently, the reaction was column-cleaned using the Zymo RNA Clean & Concentrator kit. The RNA was then decapped with 100 U mRNA Decapping Enzyme (NEB) and 40 U RiboLock at 37°C for 1 h. The reaction was column-cleaned and then denatured at 95°C for 5 min and immediately put on ice. An RNA oligo containing a 5*′* carbon spacer (5′-/5SpC3/rCrUrG rArCrA rGrCrG rArGrC rGrUrG rArGrU rUrC-3′; IDT) was ligated to the refolded RNA using T4 RNA Ligase 1 High Concentration (NEB). Refolded RNA was mixed with 40 pmol RNA oligo with 1 mM ATP, 7.5 µl of 50% PEG 8000, 3 µl of DMSO, and 45 U ligase. The reaction was incubated at 25°C for 2 h, mixed, then incubated for an additional 1 h at 25°C. An additional 15 U T4 RNA Ligase 1 High Concentration was added, and the reaction was incubated at 16°C overnight. The following day, the reaction was precipitated with 2.5 volumes, 95% ethanol; 0.1 volumes, 3 M sodium acetate, pH 5.2; and 1 µl of Glycoblue Coprecipitant (ThermoFisher). RNAs were pelleted by centrifugation at 11 000 rpm for 15 min at 4°C, washed with cold 85% ethanol, and resuspended in RNase-free water.

Resuspended RNA was then reverse transcribed with 2 pmol ZIKV-5′RACE-RT-Rev primer (5*′-*GAG CGT CAC RGC TCT TCT AGA TC-3*′*) and 0.5 mM dNTPs. The mixture was denatured at 95°C for 5 min and immediately transferred to ice, where the RT buffer and 40 U RiboLock RNase inhibitor were added. Samples were transferred to 60°C after which 200 U Maxima H Minus Reverse Transcriptase (ThermoFisher) was added. Reactions were incubated at 60°C for 1 h and then heat-inactivated at 85°C for 5 min. Template RNA hydrolysis was performed by adding 8 µl of 0.25 M EDTA/0.5 N NaOH solution and heating the reaction to 65°C for 15 min. The cDNA was column-cleaned with the Zymo DNA Clean & Concentrator kit.

cDNA was then PCR amplified using Q5 DNA polymerase (NEB) according to manufacturer’s instructions, including 5 µl of GC Enhancer (NEB), using the primers ZIKV-5RACE-PCR-GSP1v1-For (5*′-*CAG GTT CCG TAC ACA ACC CAA G-3*′*) and ZIKV-5RACE-PCR-Anchor-Rev (5*′-*CTG ACA GCG AGC GTG AGT TC*-*3′). A second PCR amplification was performed using the Q5 Hot Start High-Fidelity 2X Master Mix (NEB) according to manufacturer’s instructions with the primers ZIKV-5RACE-PCR-GSP2v1-For (5*′-*CCA TGA CCC AGC AGA AGT C*-*3′) and 5RACE-PCR-Anchor-Rev. A third PCR amplification was performed to increase yield using the same conditions as in the second PCR. The product was separated by agarose gel electrophoresis and visualized by SYBR Gold staining. The correctly sized bands were gel extracted using the QIAquick Gel Extraction Kit following each amplification. Five nanograms of gel extracted products were used as template for each PCR reaction.

The extracted band was cloned into the pUC18 plasmid vector by PCR amplifying the vector fragment using primers puc18-ZIKV-5RACE-GA-Fwd (5′-TCT GCT GGG TCA TGG TCT AGA GTC GAC CTG CAG G-3′) and puc18-ZIKV-5RACE-GA-Rev (5′-CAC GCT CGC TGT CAG GAA TTC GTA ATC ATG GTC ATA GCT G-3′). The resulting PCR product was agarose gel extracted and the insert added via Gibson assembly using the In-Fusion Snap Assembly Master Mix (Takara Bio) at a 2:1 insert:vector ratio following the manufacturer’s instructions.

The assembly reaction was then transformed into NEB 5-α Competent *E. coli* (NEB) and colonies were screened. Plasmids were extracted using the QIAprep spin miniprep kit (Qiagen) according to manufacturer’s instructions and sequenced via Sanger sequencing (Sequencing + Bioinformatics Consortium). Data shown is from three biological replicates.

### Statistical analyses

Statistical analyses were performed in GraphPad Prism 10 software. For ZIKV replicon experiments, statistical significance was determined by multiple Student’s *t* test. RNA half-lives and 95% confidence intervals were calculated by fitting the data to a one-phase exponential decay model.

## Results

### Although well conserved overall, ZIKV SLA displays distinct hotspots of sequence variability

**Figure 1.**
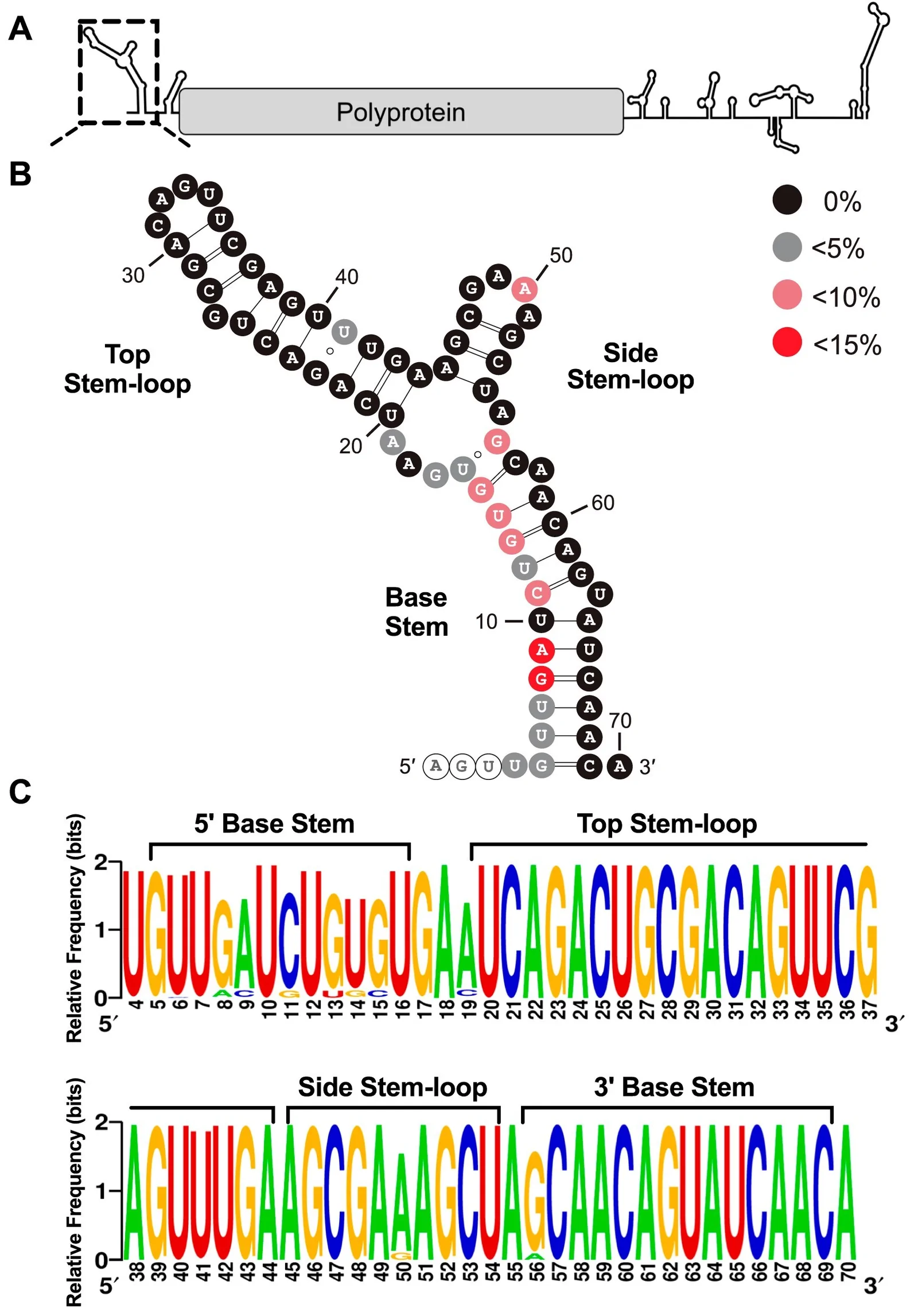
ZIKV SLA is overall well conserved but contains hotspots of variability. (**A**) Cartoon diagram of the ZIKV genome with SLA indicated (inset). (**B**) Percent variation from the consensus sequence at each nucleotide position of SLA was calculated (nts 4–70 of the ZIKV genome). Nucleotides with no variability (0%, black), <5% variability (gray), <10% variability (pink), or < 15% variability (red) are indicated. Tick marks represent 10 nucleotide intervals. (**C**) Sequence logo indicating the relative frequency of each nucleotide at each position of ZIKV SLA (nts 4–70). The base stem, top stem-loop and side stem-loop are indicated.

To explore ZIKV SLA variability, we collected ZIKV isolate sequences from the NCBI GenBank database. We retained only those sequences containing the complete SLA sequence (nts 4–70) and aligned the 97 remaining sequences, allowing us to examine nucleotide variability at each position of SLA (Fig. 1A-B). Overall, we found that SLA was highly conserved, with most positions showing no variation from the consensus sequence. In particular, the unpaired loop of top stem-loop and 3′ arm of the base stem were both highly conserved. However, we observed greater variability in the 5′ arm of the base stem as well as a few positions in the top and side stem-loops (Fig. 1B).

Given we observed several hypervariable sites, we generated a sequence logo to examine the specific nucleotide polymorphisms at each site (Fig. 1C). Interestingly, in most cases, deviations from the consensus sequence were well conserved, consistently resulting in the same patterns of sequence variability (Fig. 1C). This suggests that there are distinct hotspots for sequence variability in ZIKV SLA.

### Four distinct SLA variants cluster phylogenetically

**Figure 2.**
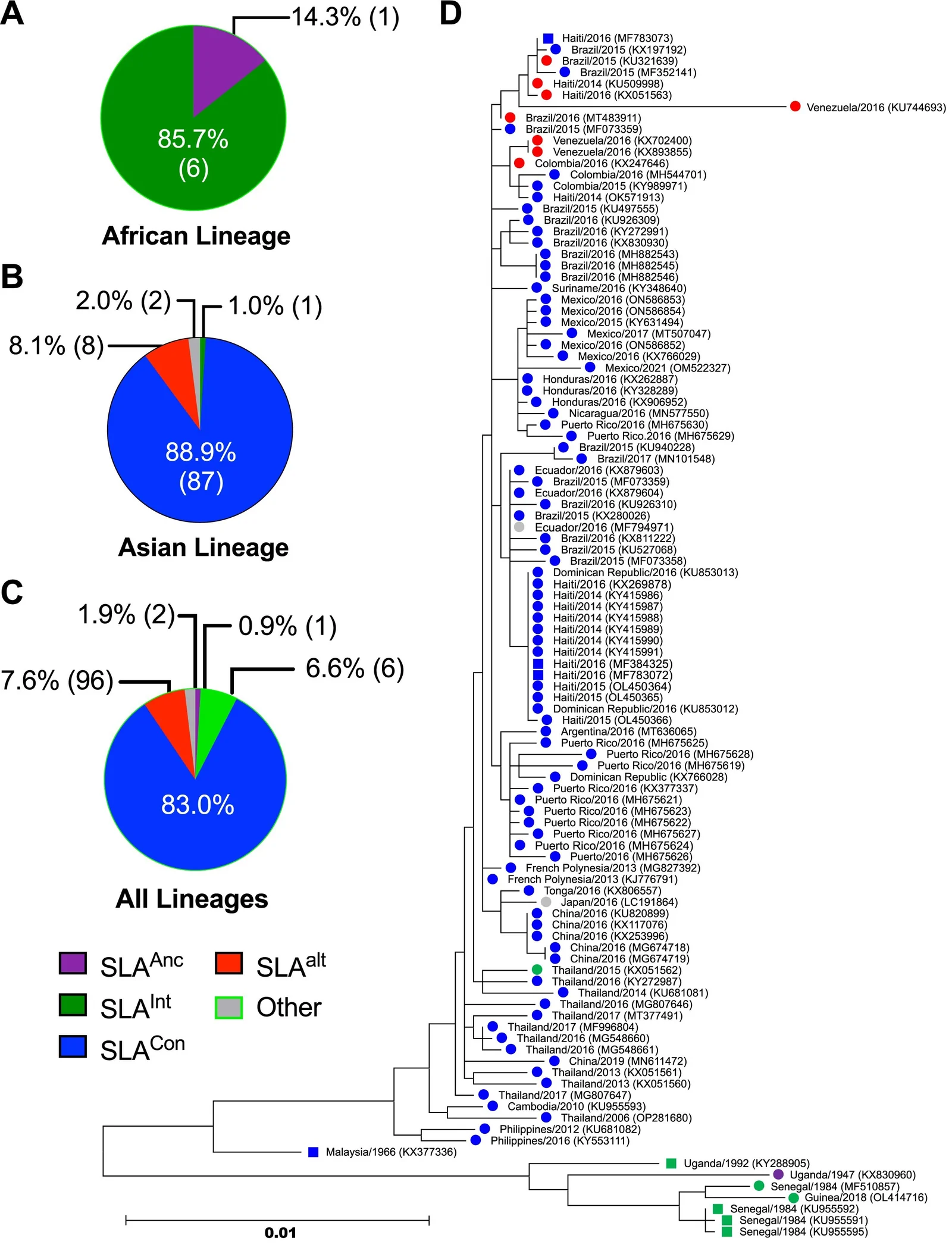
Evolutionary conservation of SLA variants. ZIKV sequences containing at least nucleotide 8 (or earlier) were used to calculate the proportion of each SLA variant across the (**A**) Asian lineage (98 sequences), (**B**) African lineage (7 sequences), or (**C**) all ZIKV sequences (105 sequences). (**D**) Maximum-likelihood phylogenetic tree of ZIKV isolates based on the complete viral polyprotein generated in MEGA11. Primate- (circle) and mosquito-derived (square) sequences as well as isolate location, year of isolation, and GenBank accession number are indicated. Colours indicate each of the SLA variants as in panels (A–C).

**Figure 3.**
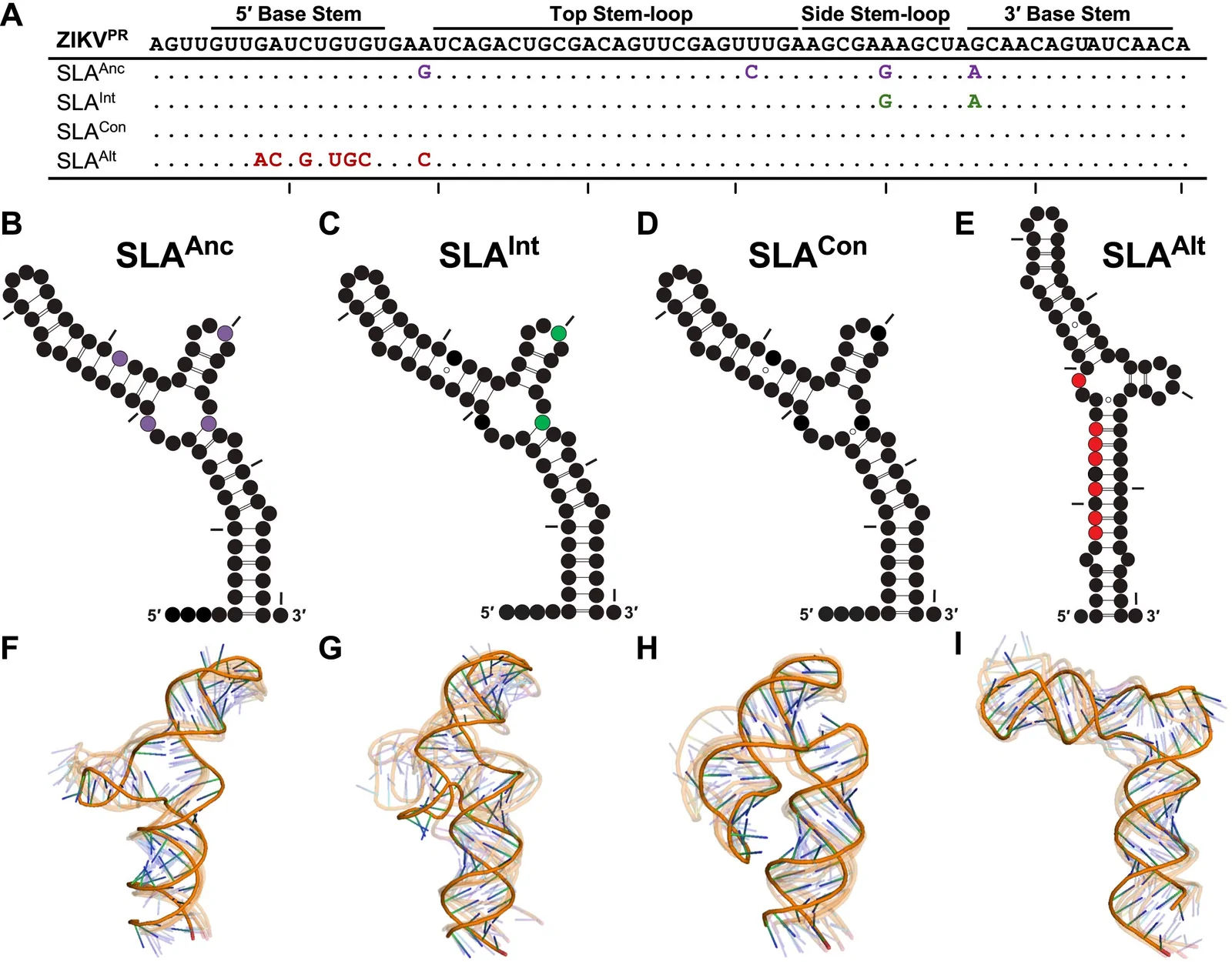
SLA nucleotide polymorphisms cluster into four distinct variants that differ in predicted structure. (**A**) Sequence alignment of identified SLA variants to the ZIKV^PR^ SLA sequence. Dots represent nucleotides identical to ZIKV^PR^, while nucleotides that differ are shown in purple (ancestral; SLA^Anc^), green (intermediate; SLA^Int^), or red (alternative; SLA^Alt^). The contemporary SLA variant (SLA^Con^) is identical to ZIKV^PR^. The most stable predicted RNA secondary structure of the four SLA variants, including: (**B**) SLA^Anc^, (**C**) SLA^Int^, (**D**) SLA^Con^, and (**E**) SLA^Alt^ are depicted. Tick marks represent 10 nucleotide intervals. RNA tertiary structure predictions of (**F**) SLA^Anc^, (**G**) SLA^Int^, (**H**), SLA^Con^, and (**I**) SLA^Alt^ generated using AlphaFold3. The prediction with the highest-ranking score is shown with full opacity, while the next four highest-ranking predicted structures are translucent.

Upon further examination, we realized that the ZIKV SLA nucleotide polymorphisms clustered phylogenetically into four distinct variants that we termed ancestral (Anc), intermediate (Int), contemporary (Con,) and alternative (Alt) (Figs 2 and 3). Given not all sequences on GenBank are complete, we compiled all unique ZIKV isolate sequences reported on GenBank that included at least nucleotide 8 of the 5′ UTR (based on alignment to a contemporary ZIKV isolate) to calculate the frequency of each SLA variant (Fig. 2A–C). We found that SLA ancestral (SLA^Anc^) represented only the ancestral Uganda 1947 MR766 ZIKV reference strain, while the remaining African lineage isolates (85.7%) contained SLA intermediate (SLA^Int^) (Fig. 2A). In contrast, the vast majority (88.9%) of the Asian lineage isolates contained SLA contemporary (SLA^Con^) (Fig. 2B). However, we noted a small cluster, representing approximately 8.1% of Asian lineage isolates, contained SLA alternative (SLA^Alt^; Fig. 2B). Interestingly, a single Asian lineage isolate from Thailand/2015 (GenBank Accession: KX051562) encoded SLA^Int^ (Fig. 2B). Overall, as the number of Asian lineage sequences available vastly outnumbers the African lineage, approximately 83% of all ZIKV sequences contain SLA^Con^, yet SLA^Int^ and SLA^Alt^ represent approximately 6.6% and 7.6% of ZIKV sequences, respectively (Fig. 2C). We also observed that two sequences contained an additional single-nucleotide polymorphism (G19, Japan 2016/LC191864) or insertion (-1G, Ecuador/2016/MF794971) that we classified as other, as they were not replicated elsewhere in our dataset and may represent either low frequency alleles or sequencing errors. However, the nucleotide polymorphisms associated with the four identified SLA variants have all been reported by multiple independent sources, and thus we chose to further characterize these four distinct SLA variants [37–41].

To better understand the phylogenetic relationship between the SLA variants, we compiled all sequences that contained the complete ZIKV coding sequence and constructed a maximum-likelihood phylogenetic tree based on the amino acid sequence of the complete polyprotein (Fig. 2D). As expected, the SLA^Anc^ and SLA^Int^ sequences clustered together in the African lineage, while SLA^Con^ appears throughout the Asian lineage across the phylogenetic tree. Additionally, independent of nucleotide sequence, all SLA^Alt^ variants clustered closely together, within the distal branches of the Asian lineage, suggesting a common origin, but they were both temporally (2014–2016) and geographically dispersed across the Caribbean and South America (Fig. 2D). Taken together, this suggests there are four distinct SLA variants that phylogenetically cluster together by lineage.

### SLA variants have alterations in predicted base pairing and/or structure

Next, we wanted to more closely examine the sequence of the SLA variants, to determine whether the nucleotide polymorphisms resulted in changes to the SLA structure, and perhaps function, in the viral life cycle. To do so, we performed a sequence alignment of the four SLA variants to a contemporary ZIKV Puerto Rican (PRVABC59) isolate (ZIKV^PR^) that we routinely use in our lab [32, 33, 42, 43]. As mentioned above, SLA^Anc^ represents the ancestral MR766 ZIKV reference strain sequence, which we found varied from the contemporary ZIKV^PR^ SLA sequence at positions 19, 41, 50, and 56 (Fig. 3A–B), while the SLA^Int^ variant differed from the ZIKV^PR^ isolate at positions 50 and 56 (Fig. 3A and C). Given SLA^Con^ represented the vast majority of Asian lineage isolates, it was unsurprisingly a perfect alignment with the contemporary ZIKV^PR^ isolate (Fig. 3A and D). Notably, the SLA^Alt^ variant contained 7 distinct nucleotide polymorphisms, at nucleotides 8–9, 11, 13–15, and 19 (Fig. 3A and E).

RNA secondary structure predictions revealed that these nucleotide polymorphisms resulted in changes in the predicted base-pairing and/or secondary structure of SLA (Fig. 3B–E). Interestingly, the polymorphisms at positions 41 and 56 in the SLA^Anc^ and SLA^Int^ variants converted Watson–Crick base-pairs to G∙U wobble base-pairs (Fig. 3B–D). Although this did not result in any major changes to the predicted SLA secondary structure, these polymorphisms correlated with a reduction in the predicted Gibbs free energy (Δ*G*) when compared with SLA^Con^ (i.e. Δ*G*^Anc^ = −24.4 kcal/mol and Δ*G*^Int^ = −21.9 kcal/mol versus Δ*G*^Con^ = −21.6 kcal/mol), suggesting that these SLA variants have a higher thermodynamic stability (Fig. 3B–D and Table 1). In these cases, only one distinct predicted SLA structure was observed. However, in the case of SLA^Alt^, the most stable predicted structure suggested that the cluster of nucleotide polymorphisms in the 5′ arm of the base stem results in a more drastic change in base-pairing, thereby lengthening the base stem and significantly increasing the overall stability of SLA^Alt^ (Δ*G*^Alt^ = −29.8 kcal/mol) (Fig. 3D–E and Table 1). Notably, SLA^Alt^ also contains fewer free nucleotides at its 5′ terminus and a slightly shorter side stem-loop when compared with the other SLA variants (Fig. 3E). Interestingly, a second alternative structure with similar Gibbs free energy (Δ*G* = −28.8 kcal/mol) was also predicted for SLA^Alt^, which displays a more “T”-shaped architecture (Supplementary Fig. S1A and B). In this structure, the unpaired sequence in the top stem-loop was shortened by one nucleotide (nts 28–30), and the AG motif, which is thought to be essential for NS5 binding, is base-paired [44]. The side stem-loop is also elongated by one base pair (nts 45–54), making its length identical to the other SLA variants, but still leaving fewer free nucleotides at its 5′ terminus. Given that the predicted Gibbs free energy is similar between the two structures (Δ*G* = −29.8 kcal/mol versus Δ*G* = −28.8 kcal/mol), it is possible that SLA^Alt^ exists in an equilibrium between these two conformations, although only the most stable predicted conformation maintains an unpaired AG motif in the top stem-loop (Table 1, and Supplementary Fig. S1A and B).

**Table 1.** Predicted Gibbs free energy and half-lives of SLA variants in cell culture

Variant	Δ*G* (kcal/mol)	Half-Life (h)	95% CIs
SLA^Anc^	−24.4	5.654	4.508–7.190
SLA^Int^	−21.9	5.101	3.383–8.286
SLA^Con^	−21.6	4.630	3.551–6.294
SLA^Alt^	−29.8 (−28.8)^a^	7.492	5.116–11.22

^a^Gibbs free energy of alternative predicted structure for SLA^Alt^

Recent structural data suggest that the three-dimensional (3D) or tertiary structure of* Orthoflavivirus* SLA may be more “V”-shaped, rather than resembling the traditionally depicted “Y”-shaped secondary structure (Figs 1 and 3B–E) [45, 46]. Thus, in addition to secondary structure predictions, we sought to perform *in silico* tertiary structure predictions of the four SLA variants (Fig. 3F–I). Like the secondary structural predictions, SLA^Anc^, SLA^Int^, and SLA^Con^ displayed a characteristic “V”-shaped tertiary structure (Fig. 3F–H). However, in line with the drastic change in secondary structure prediction, SLA^Alt^ is predicted to form a more rigid, “L”-shaped tertiary structure (Fig. 3I), resembling previously observed structures for dengue virus (DENV) SLA [44, 47]. Notably, all predicted local distance difference test (pLDDT) scores at each nucleotide were predominantly low (0.5–0.7) across the predicted tertiary structures, indicating relatively low prediction confidence. Nonetheless, secondary and tertiary structure predictions of the four SLA variants suggest that the nucleotide polymorphisms result in alterations in base-pairing and/or the overall structure of ZIKV SLA.

### *In vitro* SHAPE analysis suggests alterations in base-pairing result in changes to the overall secondary structure of the SLA variants

**Figure 4.**
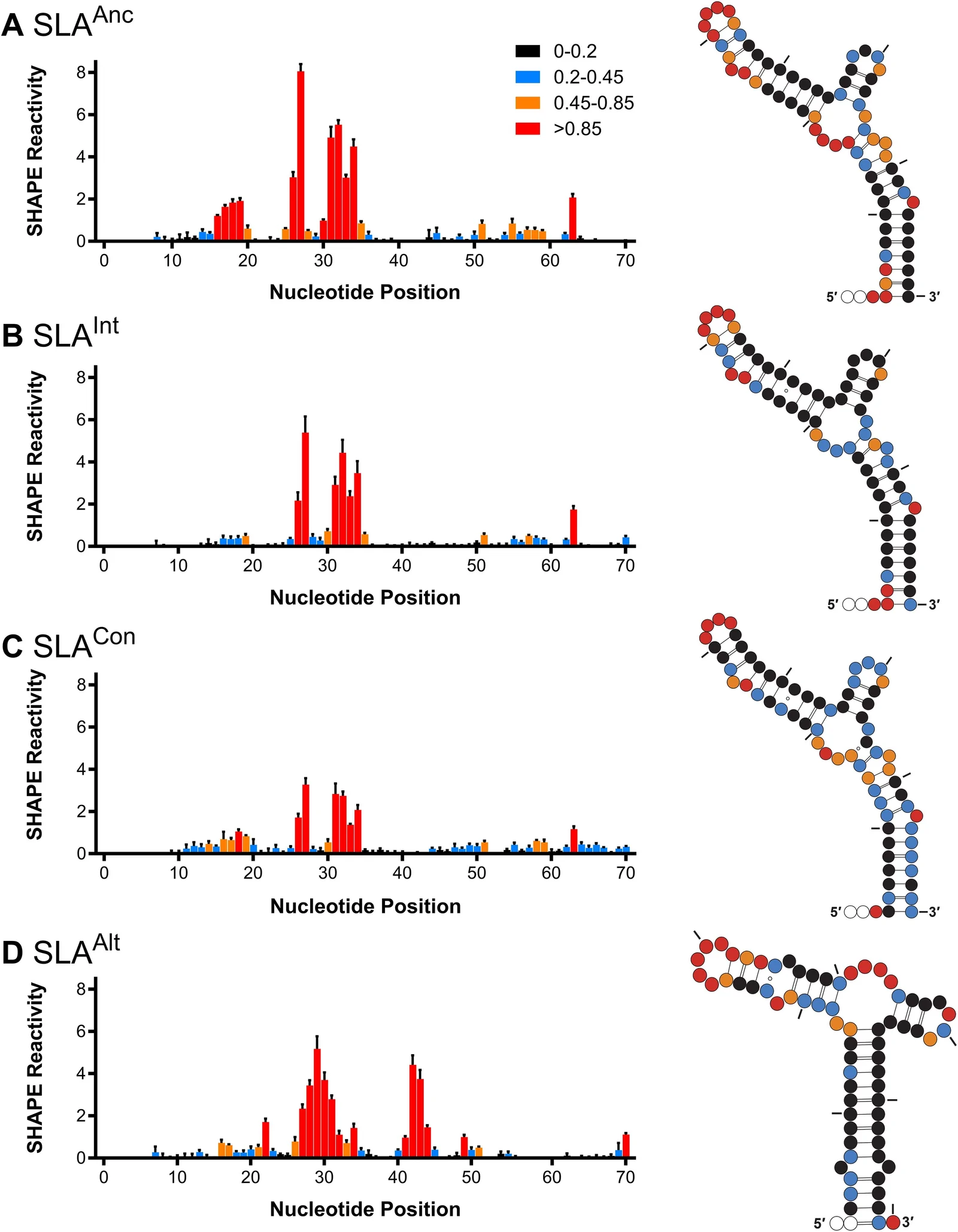
*In vitro* SHAPE analysis supports alterations in RNA base-pairing and overall secondary structure among SLA variants. Normalized selective 2´ hydroxyl acylation analyzed by primer extension (SHAPE) reactivities of nucleotides 3–70 for (**A**) SLA^Anc^, (**B**) SLA^Int^, (**C**) SLA^Con^ and (**D**) SLA^Alt^. Nucleotides with high (>0.85, red), intermediate (0.45–0.85, orange), low (0.2–0.45, blue), and very low (≤0.2, black) SHAPE reactivity are indicated. Data displayed are the SHAPE reactivity (*n* = 4) and error bars represent standard error of the mean (SEM). Prediction of the lowest free energy structures for each SLA variant constrained by SHAPE reactivity is depicted via dot plot (*right*) with relative SHAPE reactivity superimposed. Nucleotides 1–2 were omitted due to high background SHAPE reactivity (white). Tick marks represent 10 nucleotide intervals.

As our secondary structure predictions suggested changes in SLA base-pairing and/or secondary structure, we performed *in vitro* selective 2′ hydroxyl acylation analyzed by primer extension (SHAPE) to determine the secondary structure of each of the SLA variants (Fig. 4). To this end, we cloned each SLA variant into the ZIKV^PR^ backbone and performed *in vitro* SHAPE analysis followed by capillary electrophoresis on the 5′ terminus of each of the SLA variants (nts 1–383). The SHAPE results for nts 3–70 are presented in Fig. 4. Overall, the SHAPE reactivity patterns were similar for SLA^Anc^, SLA^Int^, and SLA^Con^, reflecting the similarities in their predicted secondary structures (Fig. 4A–C). Interestingly, SLA^Anc^ displayed increased SHAPE reactivity in the central bulge (nts 16–20) compared to SLA^Int^, while both SLA^Anc^ and SLA^Int^ appeared to have more reactivity than SLA^Con^ in the top stem-loop (nts 25–35), perhaps as a result of an increase in rigidity that places these nucleotides in a position to be highly reactive to the SHAPE reagent (Fig. 4A and B versus 4C) [48]. Notably, SLA^Con^ appeared to exhibit increased SHAPE reactivity across the structure, possibly suggesting a greater overall flexibility compared to the other variants.

In contrast, the SHAPE-constrained structural prediction for SLA^Alt^ suggested a novel overall architecture, presenting as a more “T”-shaped structure, more closely resembling the alternative structure predicted without SHAPE constraints (Fig. 4D and Supplementary Fig. S1). In the SHAPE-constrained prediction, there is one fewer base-pair in the base stem, and instead, one additional base-pair in the side stem-loop (nts 45–54), when compared to the unconstrained prediction. This results in a side stem-loop of the same length as the other SLA variants. Meanwhile, the base-pairing throughout the top stem-loop (nts 18–40) is distinct from the prediction (Fig. 4D and Supplementary Fig. S1). The unpaired loop in the top stem-loop in this SHAPE-constrained prediction occurs at nts 27–31, leaving the AG motif base-paired, rather than single-stranded (although this motif still exhibited high SHAPE reactivity). As discussed, the AG motif has been suggested to be crucial for NS5 binding, so it is possible that SLA^Alt^ is able to sample both of the predicted conformations, at least *in vitro* [44]. Additionally, consistent with the structural predictions, our SHAPE analysis revealed an overall reduction in labelling in the SLA^Alt^ base stem compared to other SLA variants, as well as increased labelling in the top stem-loop (nucleotides 21–44). This is reflective of the overall increase in base-pairing and lengthening of the SLA^Alt^ base stem (Fig. 4D). In contrast to the observed changes in SLA SHAPE reactivity across the four variants, we did not observe any consistent SHAPE reactivity changes between variants at any of the downstream RNA structures or circularization sequences necessary for replication, including stem-loop B (SLB), the capsid hairpin (cHP), upstream of AUG region (UAR), downstream of AUG region (DAR), or the cyclization sequence (CS) (Supplementary Fig. S2). Taken together, our SHAPE secondary structure analyses suggest that the overall secondary structure of the SLA variants is in good agreement with secondary structure predictions (Figs 3 and 4), with SLA^Anc^, SLA^Int^, and SLA^Con^ taking on a largely similar global SLA conformation, while SLA^Alt^ nucleotide polymorphisms result in a more drastic change in conformation.

### ZIKV NS5 binds to all SLA variants

**Figure 5.**
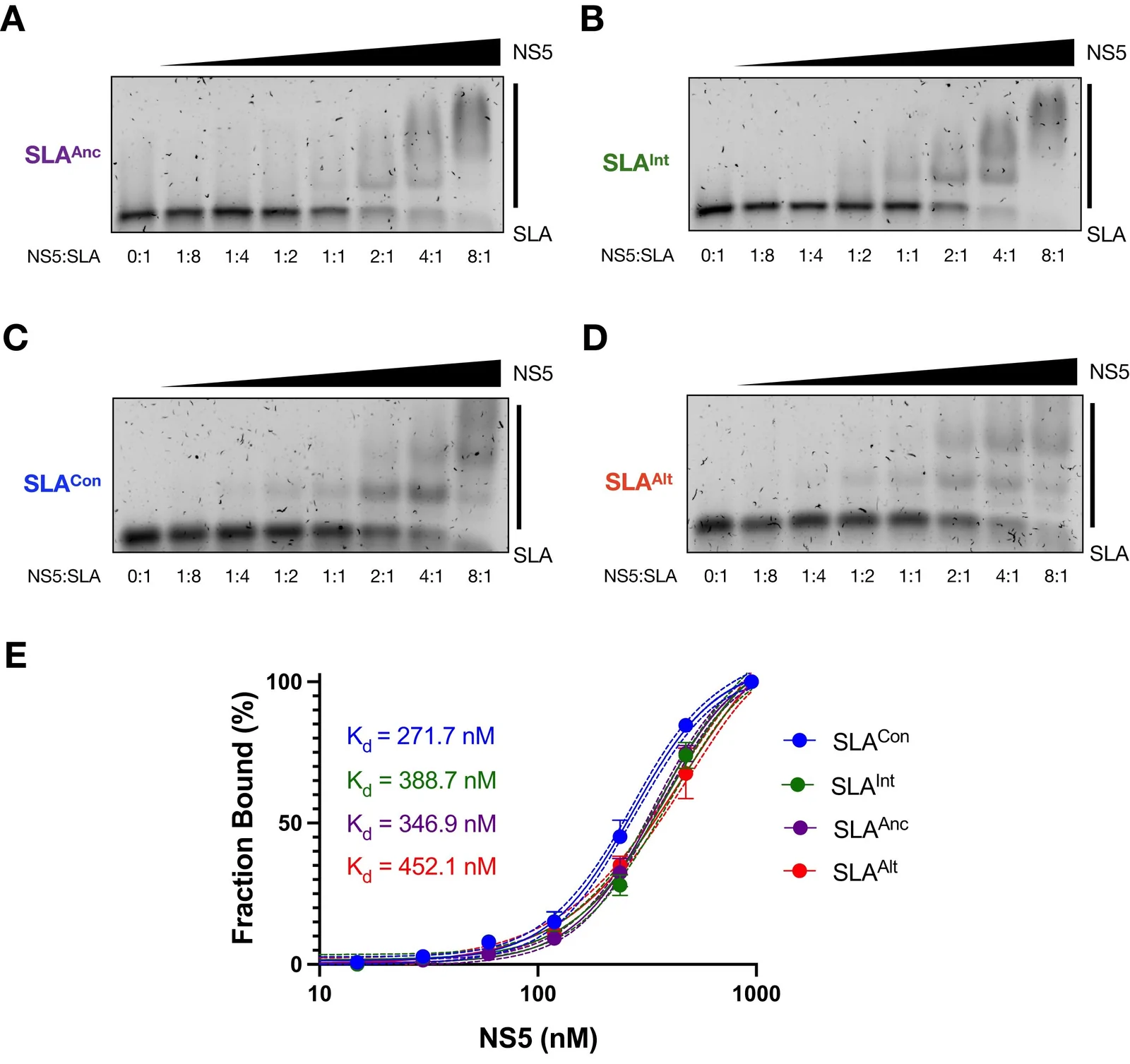
ZIKV NS5 binds to all SLA variants. EMSAs were performed using ZIKV NS5 and the first 163 nt of the ZIKV positive-strand including (**A**) SLA^Con^, (**B**) SLA^Int^, (**C**) SLA^Anc^, or (**D**) SLA^Alt^. For each RNA, 2.5 pmol (150 ng) of RNA were incubated with increasing (2-fold) amounts of NS5 ranging from 0 to 20 pmol (0–2 µg) and were then analyzed via agarose gel electrophoresis. Molar ratios of NS5:RNA are indicated. Data shown are representative of four independent replicates. (**E**) Binding curves of NS5 to each SLA-containing RNA based on the bound fraction percentage from the EMSAs in panels (A–D). Data for each RNA were fit to a sigmoidal four parameter logistic curve model. The modeled curves are depicted by solid lines for each SLA-containing RNA, with 95% confidence intervals indicated by dashed lines.

Specific binding of NS5 to SLA is thought to be mediated by interactions between the RdRp domain of NS5 and the unpaired “AG” motif in the top stem-loop of SLA [9, 19, 36, 49–54]. Since our structural and *in vitro* SHAPE analyses suggested that the architecture and dynamics of the top-stem loop of SLA differed across the variants, particularly for SLA^Alt^, we were curious whether these differences would affect their ability to bind to NS5. As such, we performed electrophoretic mobility shift assays (EMSAs) with full-length ZIKV NS5 and the 5′ terminus of the positive-strand RNA containing SLA (Fig. 5) [55]. For all four SLA variants, we observed mobility shifts with increasing molar ratio of NS5, starting at a molar ratio of around 0.5:1 (Fig. 5). Furthermore, we additionally observed multiple shifts in the EMSA at higher molar ratios of NS5:RNA, consistent with the ability of NS5 to form monomeric, dimeric, and higher-ordered oligomeric complexes *in vitro* (Fig. 5) [15, 19, 56–59]. Notably, there was no significant difference in estimated binding affinity of ZIKV NS5 between the SLA^Alt^-, SLA^Anc^-, or SLA^Int^-containing RNAs, despite SLA^Alt^ displaying a different top stem-loop architecture in our structural analyses (Fig. 5E). Conversely, despite possessing a similar structure to SLA^Anc^ and SLA^Int^, the SLA^Con^ RNA displayed a significantly higher binding affinity for ZIKV NS5 (Fig. 5E). Taken together, while all four SLA variants can bind to ZIKV NS5 *in vitro*, SLA^Con^ appears to have the highest binding affinity of the SLA variants.

### SLA variants alter viral RNA stability

**Figure 6.**
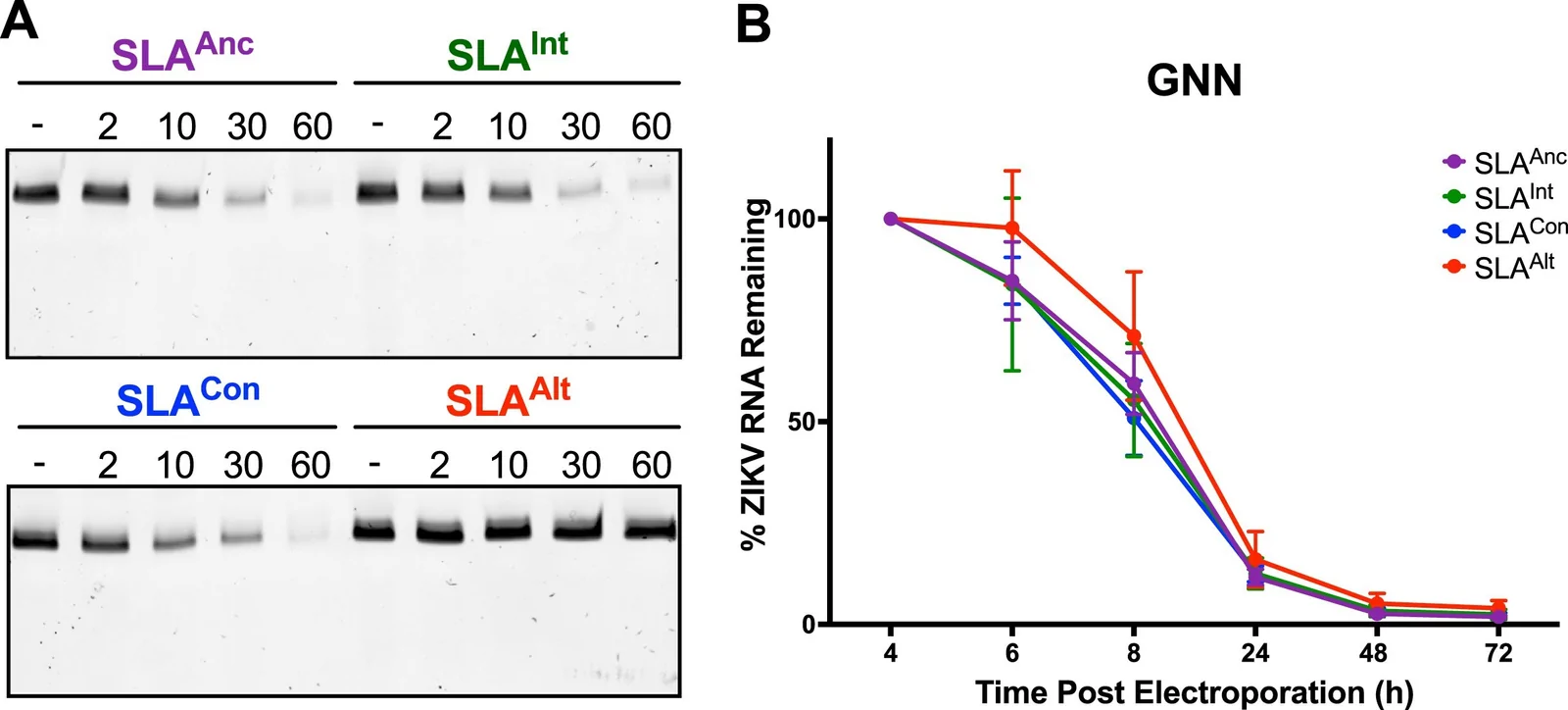
Stability of SLA variants. (**A**) 5′ exoribonuclease assay with monophosphorylated SLA RNAs (nts 1–70) incubated for 2–60 min, as indicated. RNA was visualized by denaturing urea polyacrylamide gel electrophoresis followed by SYBR Gold staining. A representative gel is shown (*n* = 3). (**B**) Replication-defective (GNN) *Renilla* luciferase (RLuc) ZIKV^PR^ replicons containing each of the SLA variants were co-electroporated with a capped Firefly luciferase (FLuc) mRNA into A549 cells. Luciferase activity was monitored over time. RLuc signal was normalized to the 2 h FLuc (electroporation control) signal; then, viral RNA decay was represented as a percentage GNN replicon RNA remaining relative to the normalized RLuc value at the 4 h time point. The data represent three independent biological replicates, and error bars represent the standard deviation (SD).

Considering our structural analyses indicated a reduction in free (unpaired) 5′ terminal nucleotides for the SLA^Alt^ variant, we hypothesized this might impact 5′ exoribonuclease resistance and viral RNA stability [31]. To examine this directly, we performed *in vitro* 5′ exoribonuclease resistance assays with each SLA variant. Interestingly, we observed that SLA^Alt^, which possesses fewer free nucleotides at its 5′ terminus and is predicted to have the lowest predicted Gibbs free energy, was completely resistant to exoribonuclease digestion at the observed timepoints (Fig. 6A). In contrast, the other SLA variants showed near-complete degradation by 60 min, suggesting that the increased base-pairing at the 5′ terminus and/or the increased Gibbs free energy provides protection of the SLA^Alt^ variant from 5′ exoribonuclease activity.

We then wondered if this inherent stability of SLA^Alt^ might alter viral RNA stability in cell culture by modulating the ability of decapping enzymes and/or exoribonucleases from accessing the 5′ terminus of the viral RNA [31, 60, 61]. To explore this possibility, we introduced each SLA variant into a ZIKV^PR^  *Renilla* luciferase subgenomic replicon backbone, which canonically encodes SLA^Con^. We used a replication-defective replicon, containing inactivating mutations in the NS5 RdRp active site (GDD → GNN) to assess subgenomic replicon RNA stability in the absence of viral RNA replication. Briefly, IVT replication-defective (GNN) subgenomic replicon RNAs containing each of the SLA variants were introduced into cells via electroporation and luciferase activity was monitored from 2 to 72 h post-electroporation. We used these data to approximate the half-lives of each of the SLA variants in cell culture (Fig. 6B and Table 1). Interestingly, while the results did not reach statistical significance, we observed that the half-lives appeared to correlate with the ΔG predictions for each of the variants, with SLA^Alt^ exhibiting the longest half-life, followed by SLA^Anc^, SLA^Int^, and SLA^Con^ (Table 1). Taken together, our results suggest that the increase in base-pairing at the 5′ terminus of SLA^Alt^ increases viral RNA stability both *in vitro* using 5′ exoribonuclease assays and, while it did not reach statistical significance, we also observed a similar trend in cell culture.

### SLA variants alter viral RNA accumulation in a subgenomic replicon model

**Figure 7.**
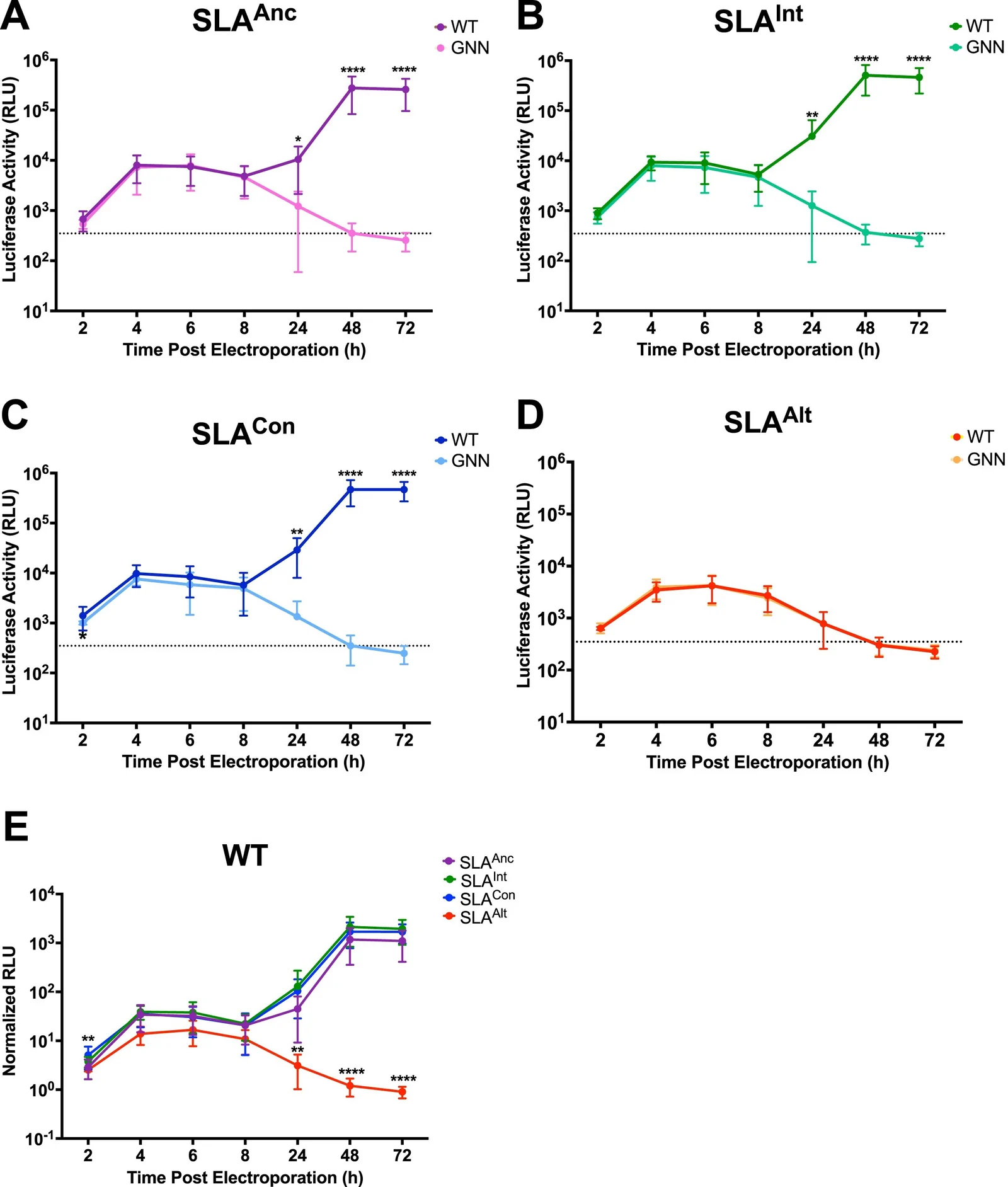
SLA variant accumulation in mammalian cell culture. Replication-competent (WT) or replication-defective (GNN) RLuc ZIKV^PR^ replicons containing each of the SLA variants were co-electroporated with a capped Firefly luciferase (FLuc) mRNA into A549 cells. Luciferase activity was measured at the indicated time points post-electroporation. Raw luciferase activity of individual SLA variants, including (**A**) SLA^Anc^, (**B**) SLA^Int^, (**C**), SLA^Con^, and (**D**) SLA^Alt^ is shown. Error bars represent the SD, and the limit of detection is indicated via a dashed line. (**E**) Combined data for the replication-competent variants normalized to the 2 h FLuc mRNA signal (electroporation efficiency control). Significance of SLA^Alt^ compared to SLA^Con^ is shown, and error bars represent the SD. All data represent three independent biological replicates. *P < 0.05, **P < 0.01, ***P < 0.001, ****P < 0.0001.

Given the importance of SLA in orthoflavivirus RNA replication, we sought to assess the effect of these nucleotide polymorphisms on viral RNA accumulation in cell culture in a replication-competent context. To this end, IVT replication-competent (wild-type, WT), as well as replication-defective (GNN) subgenomic replicon RNAs, containing each of the SLA variants were introduced into mammalian (A549) and mosquito (Aag-2) cells via electroporation and luciferase activity was monitored for several days post-electroporation (Fig. 7 and Supplementary Fig. S3). At early timepoints (2–8 h), we observed that all SLA variants were translationally-competent, although the SLA^Alt^ variant appeared to have a slight impairment in translation, which was statistically significant at the 2 h timepoint, but not at later timepoints (Fig. 7A–E). In contrast, at late timepoints post-electroporation (24–72 h), while SLA^Anc^, SLA^Int^, and SLA^Con^ viral RNAs accumulated to similar extents, we did not observe any RNA accumulation in SLA^Alt^ (Fig. 7A–E). A similar pattern was observed in the mosquito (Aag-2) cells over a 7-day time course. Taken together, these results suggest that while SLA^Anc^, SLA^Int^, and SLA^Con^ are all replication-competent in the ZIKV^PR^ background, the introduction of SLA^Alt^ into the ZIKV^PR^ background is lethal for viral RNA accumulation.

As we were perplexed by the inability of SLA^Alt^ to support viral RNA accumulation in the ZIKV^PR^ background, we made several attempts to rescue viral RNA replication of the SLA^Alt^-containing replicon RNA. First, given SLA^Alt^ appeared to have a translation defect (Fig. 7E), we attempted to boost viral protein levels via *trans*-complementation, through co-electroporation of a SLA^Con^ GNN (replication-defective) replicon RNA with the SLA^Alt^ replicon RNA. However, we still did not observe SLA^Alt^ RNA accumulation, suggesting that the defect is not solely attributable to a defect in viral translation, or that the defect is not amenable to *trans*-complementation (Supplementary Fig. S4A). Second, we hypothesized that SLA^Alt^-encoding strains may encode unique amino acids, particularly within the NS5 gene, that could have evolved to accommodate SLA^Alt^.

We thus aligned the amino acid sequence of SLA^Alt^-encoding strains against the full-length ZIKV^PR^ amino acid sequence and identified the V2611A amino acid polymorphism that was present in the NS5 region of all SLA^Alt^-encoding strains, but absent from our parental ZIKV^PR^ replicon (Supplementary Table S2). Thus, we cloned this amino acid mutation into our subgenomic replicon system encoding SLA^Alt^. However, we still did not observe any SLA^Alt^ RNA accumulation in this context (Supplementary Fig. S4B). Collectively, these results suggest that evolutionarily acquired nucleotide polymorphisms in SLA alter its ability to promote viral RNA accumulation in cell culture.

### 5′ RACE reveals a novel SLA^Alt^-encoding ZIKV isolate

**Figure 8.**
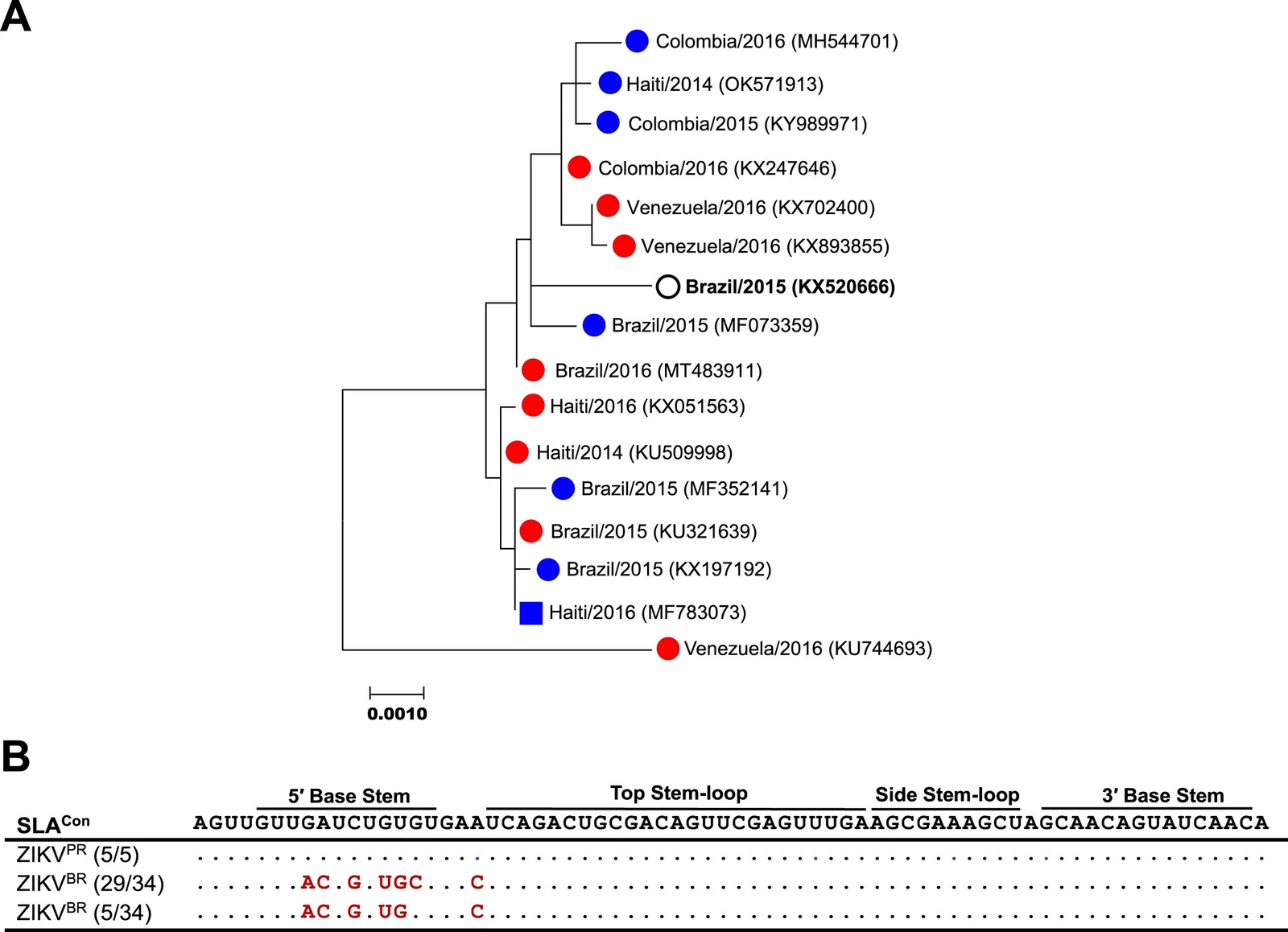
5′ Rapid amplification of cDNA ends (RACE) analysis of Asian lineage isolates. (**A**) Maximum-likelihood phylogenetic tree of a subset of ZIKV isolates constructed in MEGA11. ZIKV^BR^ (HS-2015-BA-01, GenBank Accession: KX520666) is in bold and denoted with an open circle. (**B**) 5′ RACE analysis was performed on total RNA after infection (MOI = 3) of Huh-7.5 cells with two Asian lineage isolates from Puerto Rico, ZIKV^PR^ (PRVABC59, GenBank Accession: KX377337) and Brazil, ZIKV^BR^. Sequences were aligned to the SLA^Con^ consensus sequence, and the total number of clones sequenced are indicated for each isolate.

Despite our inability to rescue replication of the SLA^Alt^ variant in the ZIKV^PR^ subgenomic replicon system, SLA^Alt^ has been observed several times in nature (Fig. 2). Interestingly, phylogenetic analysis of a Brazilian ZIKV isolate (ZIKV^BR^, HS-2015-BA-01, GenBank Accession: KX520666) in our lab revealed that it clustered with other SLA^Alt^-containing viruses, although the identity of its 5′ terminus was unclear, as the sequence deposited on GenBank was incomplete (Fig. 8A) [42]. ZIKV^BR^ was isolated in Salvador, Bahia (Brazil) in 2015 from the blood of an infected female patient [62]. Thus, to determine the identity of ZIKV^BR^ SLA, we performed 5′ RACE analysis after infection with the ZIKV^PR^ and ZIKV^BR^ isolates, with the former’s complete genome sequence already known (Fig. 8). As expected, for ZIKV^PR^, all 5 clones sequenced contained the SLA^Con^ variant; however, of the 34 clones sequenced for ZIKV^BR^, 29 contained SLA^Alt^, while 5 contained SLA^Alt^ with an additional single-nucleotide polymorphism, C15G, a polymorphism present in SLA^Con^ (Fig. 8B). Given this single-nucleotide polymorphism, we next tested whether this mutation could confer RNA accumulation when introduced into our subgenomic replicon model. However, our results suggest that like the SLA^Alt^ variant, SLA^Alt^ C15G was also unable to accumulate in cell culture (Supplementary Fig. S5). Additionally, we performed 3′ RACE analysis on the ZIKV^BR^ isolate to examine any compensatory mutations that might be present in the viral 3′ UTR. But, consistent with the other published sequences of SLA^Alt^-containing viruses, we did not observe any nucleotide polymorphisms in this region compared with ZIKV^PR^ (data not shown). As such, our data confirm that SLA^Alt^ is present in a natural context and is found in a commonly used 2015 Brazilian isolate (HS-2015-BA-01) that phylogenetically clusters with other SLA^Alt^-encoding isolates. Given that this isolate has been widely used in both cell culture and animal studies, and was passaged herein, the SLA^Alt^ variant is clearly capable of promoting viral RNA accumulation in the context of this ZIKV^BR^ isolate [42, 62–71]. Future studies will thus be focused on determining the contextual differences that facilitate SLA^Alt^-driven viral RNA accumulation.

## Discussion

Herein, we have characterized the evolutionary landscape of the ZIKV 5′ UTR, identifying four distinct SLA variants (SLA^Anc^, SLA^Int^, SLA^Con^, and SLA^Alt^) and their impact on NS5 binding, RNA stability, and viral RNA accumulation. While the essential motifs for NS5 recognition—the top stem-loop AG motif and the U-bulge of the base stem—remain 100% conserved, hotspots of variability in the base stem and central bulge fundamentally alter the mechanical flexibility and thermodynamic stability of the promoter.

### Evolutionary selection for promoter accessibility

Our analysis revealed a clear lineage-based clustering of SLA variants, suggesting that the transition from the African lineage (SLA^Anc^ and SLA^Int^), to the contemporary Asian lineage (SLA^Con^) represents a strategic evolutionary shift. Notably, SLA^Con^—which dominates recent global outbreaks—exhibits a higher binding affinity for ZIKV NS5 compared to ancestral forms, potentially streamlining the transition to replication. Interestingly, the SLA^Alt^ variants cluster most distally to the Asian lineage, representing a marked structural divergence not only from the ancestral strains but also from the contemporary consensus.

This divergent “closed” architecture, characterized by additional base-pairing at the 5′ terminus, confers superior resistance to host 5′ exoribonuclease activity and may ultimately enhance RNA longevity. However, our data suggest that this stability may function as a double-edged sword. This “closed” architecture could potentially restrict access to the 5′ cap by host RNA binding proteins or impair eIF4A-mediated unwinding during translation initiation. Furthermore, the rigidified fold may impose a kinetic barrier to the recruitment of the replication and capping machinery.

The dominance of SLA^Con^ in recent outbreaks suggests an evolutionary preference for kinetic accessibility—prioritizing replicative fitness over the inherent stability of the individual RNA molecule. In this context, SLA^Alt^ appears to represent a specialized evolutionary trajectory that may prioritize genome persistence, potentially at the expense of the kinetic accessibility that characterizes contemporary pandemic strains. Ultimately, this lineage-specific partitioning illustrates how the structural evolution of RNA serves as a key determinant of viral fitness and the emergence of distinct, isolate-specific replication strategies.

### The SLA^Alt^ paradox: structural plasticity and genetic epistasis

A pivotal finding of this study is the identification of SLA^Alt^, a variant previously dismissed as a sequencing artifact [72]. We independently confirmed the existence of this variant via 5′ RACE of a clinical isolate from Brazil (ZIKV^BR^, HS-2015-BA-01). Despite its presence in nature, SLA^Alt^ was lethal when introduced into the contemporary ZIKV^PR^ backbone.

The replication-defective phenotype observed of this variant in a non-native context highlights a high degree of genetic epistasis. Indeed, our results suggest that the SLA architecture is not a static scaffold, but a dynamic element that must co-evolve with its genomic environment. Notably, we identified three conserved amino acid substitutions in ZIKV^BR^ (capsid T80I, NS1 GH892W, and NS5 V2611A) that may serve as compensatory markers; however, the NS5 V2611A mutation alone was insufficient to rescue replication. Additionally, previous reports suggest that the ZIKV 5′ UTR, including sequences in SLA, may form functional long-range RNA–RNA interactions with the envelope (E) coding region and the terminal 3′ stem-loop region of the genome [73, 74]. However, the consensus sequence and our 3′ RACE analyses performed herein suggest that the ZIKV^BR^ isolate has 100% sequence identity to ZIKV^PR^ in these regions, suggesting that disruption of these interactions is not responsible for the defect in SLA^Alt^ viral RNA accumulation in the context of the contemporary subgenomic replicon backbone. Taken together, our fundings suggest a lock-and-key model, where specific protein–RNA or RNA–RNA interactions (i.e. during cyclization) may be required to accommodate this rigidified SLA^Alt^ structure. Intriguingly, similar variation in SLA sequence and structure has been observed in the related orthoflaviviruses, including dengue and Japanese encephalitis virus, suggesting that variability in SLA may be selected for across numerous orthoflavivirus species [75, 76]. This suggests that the structural plasticity observed in ZIKV is not an isolated phenomenon, but rather a conserved mechanism of viral adaptation.

### Functional implications for ZIKV persistence

The increased stability of SLA^Alt^ may explain the unique phenotypes of the ZIKV^BR^ isolate, which has been shown to induce reduced CD8+ T-cell responses and exhibit greater persistence *in* vivo [67, 68]. Indeed, we previously demonstrated that in cell culture, ZIKV^BR^ displays increased dsRNA accumulation and significantly higher viral titers over a single infectious cycle compared to ZIKV^PR^ [42]. This observation aligns with the increased thermodynamic stability and 5′ exoribonuclease resistance we observed herein.

By protecting the 5′ terminus from the host decay machinery, SLA^Alt^ may facilitate a “slow and steady” replication strategy; while the rigidified structure may impose a higher kinetic barrier to initiation, the enhanced longevity of the genomic template likely supports a more robust accumulation of viral progeny over the course of the infection. This strategy may allow ZIKV^BR^ to evade host immune detection more effectively than the more “accessible” SLA^Con^ variants. However, this stability could come at the cost of sfRNA production, which is known for antagonizing interferon responses [24, 77]. This delicate balance between genome protection and sfRNA-mediated immune evasion likely dictates the adaptive trajectory of emerging ZIKV clades.

### Limitations and future directions

While our study provides a detailed characterization of SLA evolution, several limitations remain. First, our structural probing, EMSA analysis, and exoribonuclease assays were conducted *in vitro* using purified proteins and RNA; therefore, we cannot fully account for the influence of additional host or viral RNA-binding proteins or the specific microenvironment of the viral replication organelle, which may further modulate the SLA conformation *in vivo*. Additionally, our functional assays primarily utilized a subgenomic replicon system in a single genetic backbone (ZIKV^PR^). While this allowed us to isolate the effects of SLA polymorphisms, it may not fully capture the synergistic effects of coding-region mutations or the nuances of a multi-cycle infection in a full-length infectious clone. Finally, the lack of available metadata and the inherent sampling biases in public sequence databases mean that additional, low-frequency SLA variants may exist, particularly within the under-sampled African lineage. Future studies using a wider array of clinical isolates and native genomic backbones will be important to fully map the epistatic network that governs the ZIKV promoter plasticity.

## Conclusions

Our findings demonstrate that despite strong sequence conservation in essential motifs, ZIKV SLA has evolved at least four distinct structural variants. The discovery of the SLA^Alt^ variant—which enhances RNA stability *in vitro* yet impairs accumulation in contemporary replicon systems—highlights the critical importance of considering evolutionarily acquired nucleotide polymorphisms within viral UTRs. This work supports the growing consensus that evolutionary pressures act not only on viral coding sequences but also within non-coding regions to calibrate viral fitness. Ultimately, this study underscores the indispensable role of SLA in viral RNA accumulation and provides a more nuanced understanding of how RNA structure and function drive the orthoflavivirus life cycle.

## Supplementary Material

ugag027_Supplemental_File

## Data Availability

The data underlying this article will be shared on reasonable request to the corresponding author.
