# Correction to ‘Evolutionary refinement of the ZIKV 5′ UTR dictates a trade-off between RNA stability and promoter accessibility’

**DOI:** 10.1093/narmme/ugag042

**Published:** 2026-09-25

This is a correction to: Alex B Wang, Quinn H Abram, Carolina Camargo, Trisha R Barnard, Selena M Sagan, Evolutionary refinement of the ZIKV 5′ UTR dictates a trade-off between RNA stability and promoter accessibility, *NAR Molecular Medicine*, Volume 3, Issue 2, April 2026, ugag027, https://doi.org/10.1093/narmme/ugag027

In the originally version of the paper, an erroneous duplication of images was published in Figure 5 which should read:


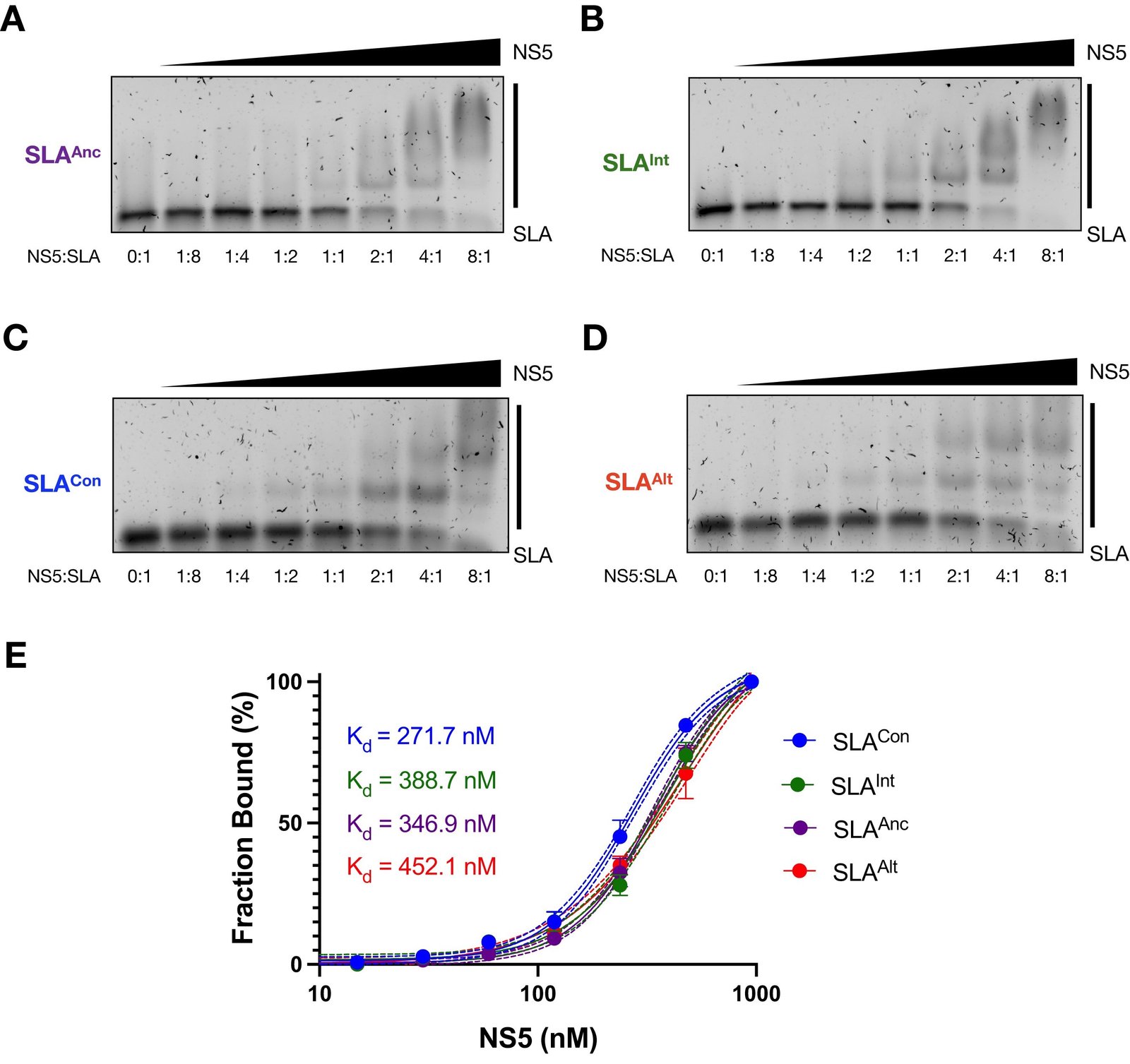


instead of:


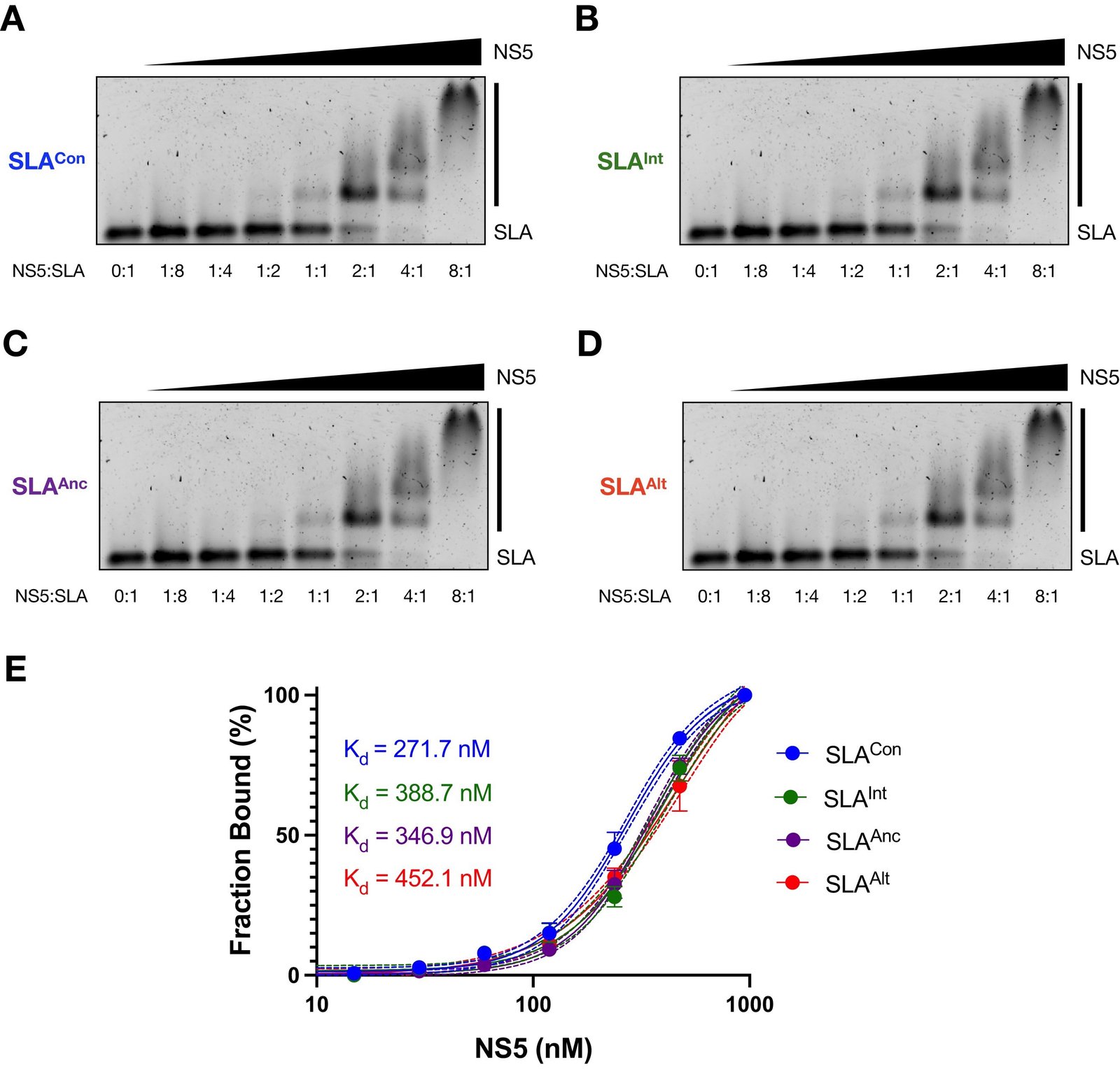


The emendation has been made to the paper.

