# Supplementary material for: Evolutionary refinement of the ZIKV 5′ UTR dictates a trade-off between RNA stability and promoter accessibility
Source: NAR Mol Med. 2026 May 22;3(2):ugag027. doi: 10.1093/narmme/ugag027 (PMC13221642; doi:10.1093/narmme/ugag027)
Supplement: ugag027_Supplemental_File [file ugag027_supplemental_file.pdf]

Supplementary Methods

3′ Rapid Amplification of cDNA Ends (RACE) Analysis

Total RNA was extracted from cells using TRIzol Reagent according to manufacturer's instructions. Ten micrograms of RNA was treated with 5 U *E. coli* Poly(A) Polymerase (NEB), supplemented with 40 U Ribolock, according to manufacturer's instructions. The reaction was column-cleaned using the Zymo RNA Clean & Concentrator kit and eluted in RNase free water.

RNA was then reverse transcribed with 2 pmol ZIKV-3′RACE-RT-Rev tagged poly(dT) primer (5′- CGC GCG GTC CGC CGG GTA GAATTC TTT TTT TTT TTT TTT TTT TT-3′) and 0.5 mM dNTPs. The mixture was denatured at 95°C for 5 minutes (min) and immediately transferred to ice, where the RT buffer and 40 U RiboLock RNase inhibitor were added. Samples were transferred to 50°C after which 200 U Maxima H Minus Reverse Transcriptase (ThermoFisher) was added. Reactions were incubated at 50°C for 30 mins and then heat-inactivated at 85°C for 5 min. Template RNA hydrolysis was performed by adding 8 μL of 0.25 M EDTA/0.5 N NaOH solution and heating the reaction to 65°C for 15 min. The cDNA was precipitated as described above and resuspended in 10 μL RNase free water.

Five microliters of cDNA was then PCR amplified using Q5 Hot Start High-Fidelity 2X Master Mix and the primers ZIKV-3RACE-PCR-GSP1v1-For (5′- TGG ATG GGG AGA GAG AAC TCA GGA GGT GGT GTT G -3′) and ZIKV-3RACE-PCR-Rev (5′- CGC GCG GTC CGC CGG GTA GAA TTC -3′). A second PCR amplification was similarly performed using the Q5 Hot Start High-Fidelity 2X Master Mix (NEB) using 10% of the first reaction volume with the primers ZIKV-3RACE-PCR-GSP2v2-For (5′- CAC AGT CAA CAT GGT GCG CAG GAT CAT AGG TGA TGA AG -3′) and ZIKV-3RACE-PCR-Rev. The product was separated by agarose gel electrophoresis and visualized by SYBR Gold staining. The correctly sized bands were gel extracted using the QIAquick Gel Extraction Kit following the second amplification.

The extracted band was cloned into the pUC18 plasmid vector by PCR amplifying the vector fragment using primers puc18-ZIKV-3RACE-GA-Fwd (5′- ACC ATG TTG ACT GTG GGT CGG CATG GCA TCT CCA C -3′) and puc18-ZIKV-3RACE-GA-Rev (5′- CCG GCG GAC CGC GCG GTA ATC ATG GTC ATA GCT GTT TCC -3′). The resulting PCR product was agarose gel extracted, and the insert added via Gibson assembly using the In-Fusion Snap Assembly Master Mix (Takara Bio) at a 2:1 insert:vector ratio according to manufacturer's instructions.

The assembly reaction was then transformed into NEB 5-alpha Competent *E. coli* (NEB) and colonies were screened. Plasmids were extracted using the QIAprep spin miniprep kit (Qiagen) according to manufacturer's instructions and sequenced via Sanger sequencing (Sequencing + Bioinformatics Consortium).

Biological Resources

Aedes aegypti (Aag2) cells were maintained in Schneider's insect media with 10% fetal bovine serum (FBS), 1% nonessential amino acids and 1% L-glutamine (Schneider's complete media). Cells were maintained at 28° in a humidified atmosphere without CO_2 and were routinely screened for mycoplasma contamination.

Aag2 electroporation

Aag2 cells were trypsinized and washed twice with cold phosphate-buffered saline (PBS; Gibco). Eight million cells were resuspended in 800 µL of cold PBS, mixed with 5 µg capped ZIKV subgenomic replicon IVT RNA, and 1 µg capped and polyadenylated FLuc mRNA (electroporation control) in a cold 4-mm electroporation cuvette. Cells were electroporated with three consecutive exponential pulses at 850 volts (V), 25 µF, and infinite resistance, optimized for the Bio-Rad Gene Pulser XCell (Bio-Rad). After a 10 min recovery at room temperature, the electroporated cells were resuspended in Schneider's complete media and 2 mL per timepoint were plated in either 15 mL Falcon tubes (for 2-8 h timepoints), 12-well (day 1 timepoint) or 6-well plates (day 2-7 timepoints).

Supplementary Figures

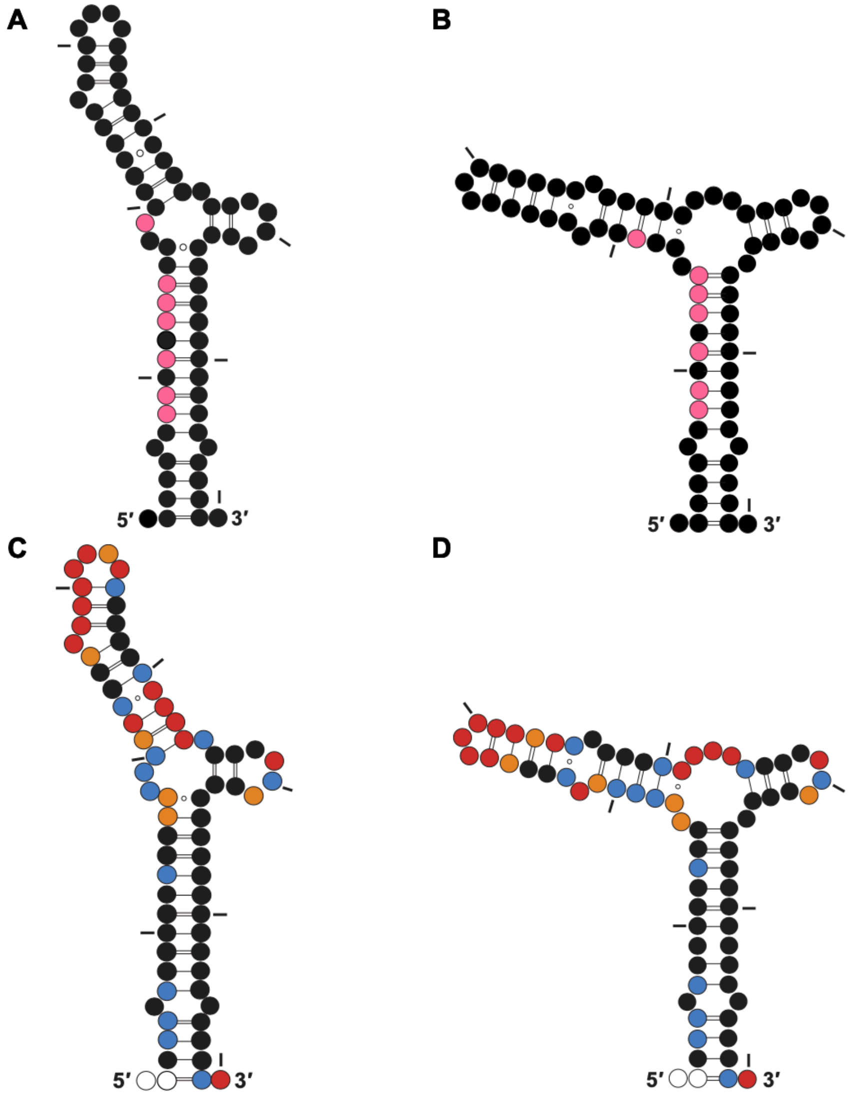

Figure S1. Alternative secondary structure prediction of SLA^{Alt}. (**A**) Most stable and (**B**) alternative (second most stable) predicted RNA secondary structure of SLA^{Alt}, with SLA^{Alt}-associated polymorphisms indicated in pink. Normalized selective 2´ hydroxyl acylation analyzed by primer extension (SHAPE) reactivities of nucleotides 3-70 overlaid over the (**C**) most stable and (**D**) second most stable predicted RNA secondary structure of SLA^{Alt} when predicted without SHAPE constraints. Nucleotides with high (>0.85, red), intermediate (0.45-0.85, orange), low (0.2-0.45, blue), and very low (≤ 0.2, black) SHAPE reactivity are indicated (n = 4). Nucleotides 1-2 were omitted due to high background SHAPE reactivity (white). Ticks indicate 10 nucleotide intervals.

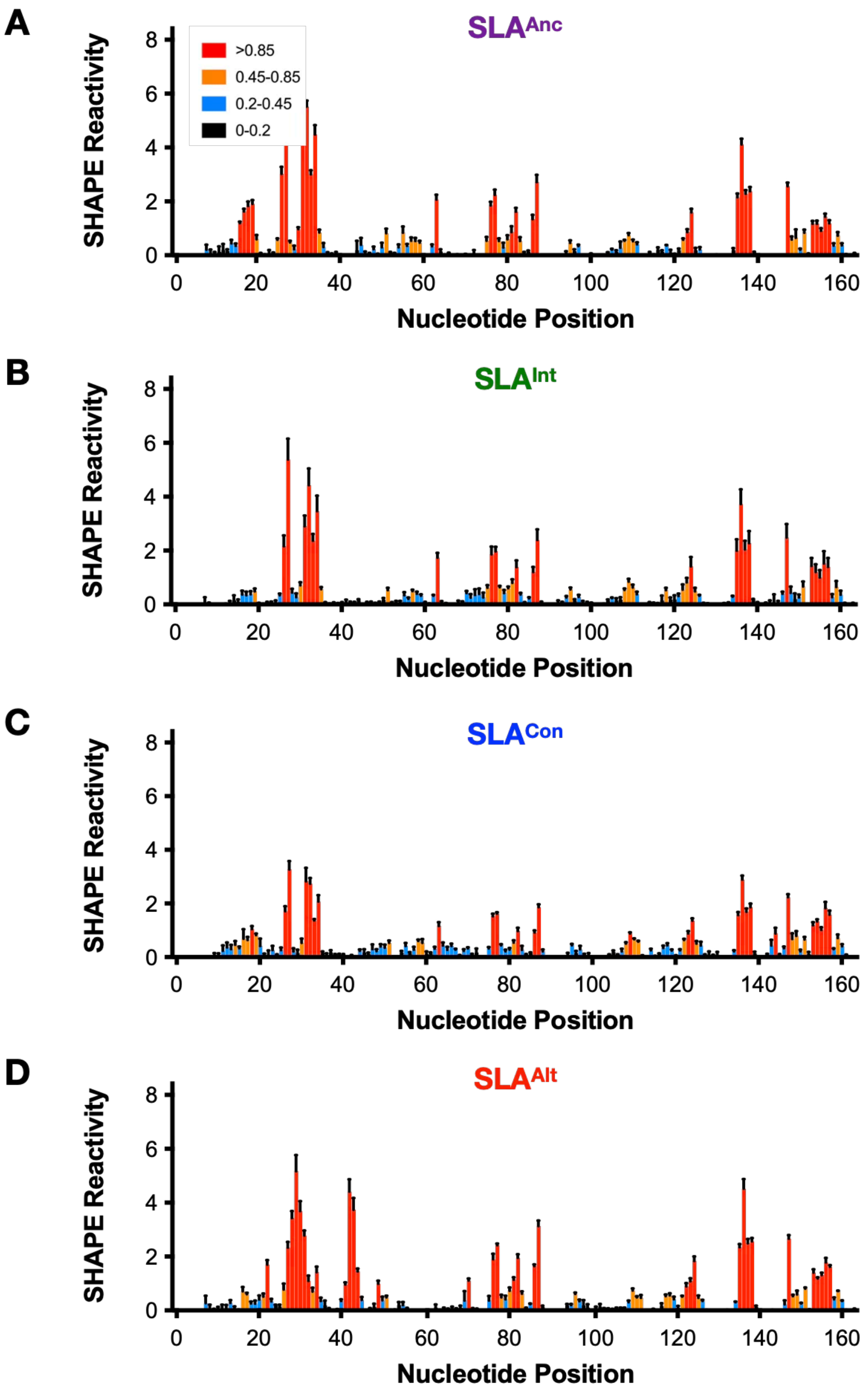

Figure S2. SLA variants do not induce changes in SHAPE reactivity in downstream structures. (A-D) Normalized SHAPE reactivities of the first 163 nt at the 5′terminus of the positive-strand of ZIKV for (A) SLA^{Con}, (B) SLA^{Int}, (C) SLA^{Anc}, or (D) SLA^{Alt}. Data is shown as the normalized SHAPE reactivity from four biological replicates and error bars represent the SEM. Nucleotides with very low (≤ 0.2), low (0.2 - 0.4, blue), intermediate (0.4 - 0.85, orange), and high (≥ 0.85, red) SHAPE reactivity are indicated. Nucleotides 1-6 were omitted due to high background reactivity.

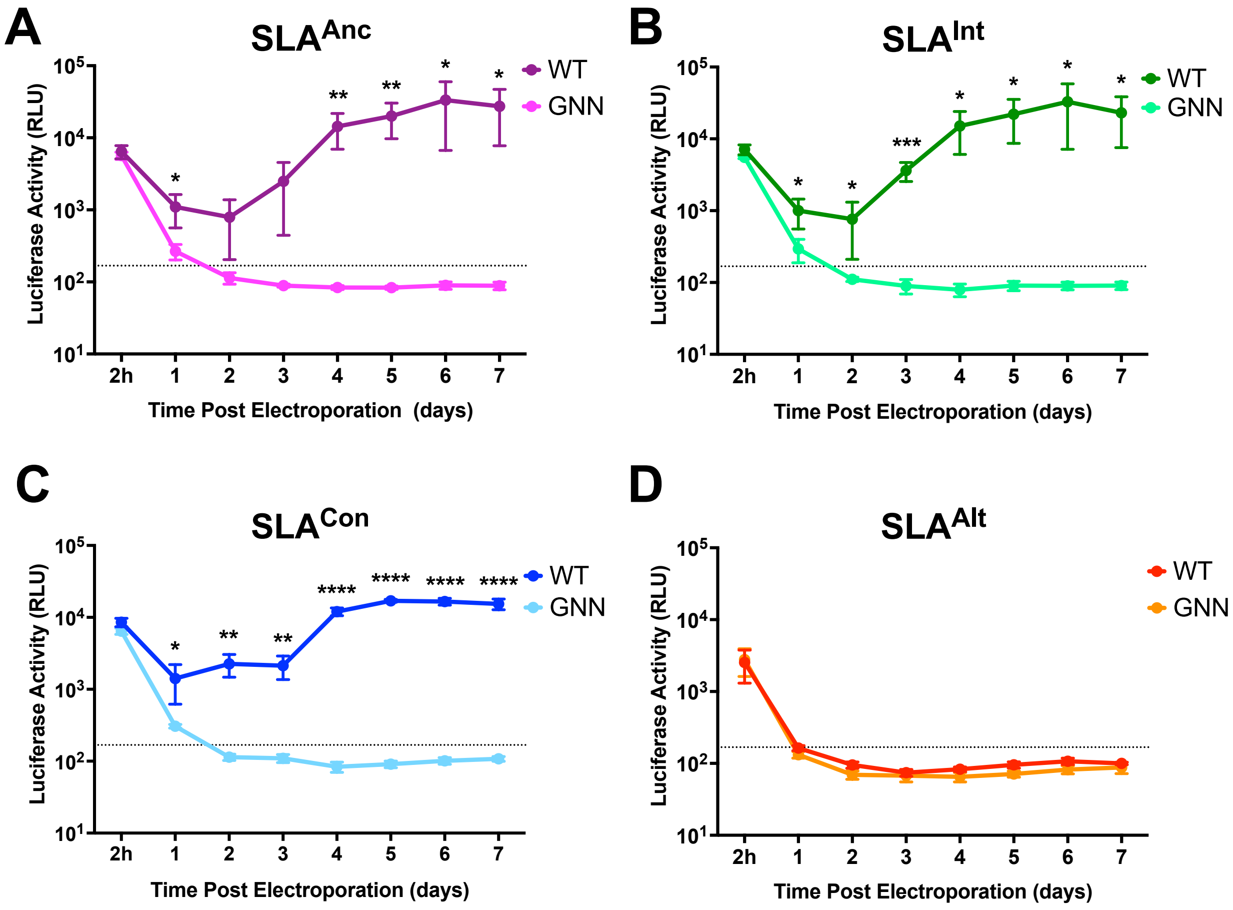

Figure S3. SLA variant accumulation in mosquito cell culture. Replication-competent (WT) or replication-defective (GNN) RLuc ZIKV^{PR} replicons containing each of the SLA variants were co-electroporated with a capped Firefly luciferase (FLuc) mRNA into Aag-2 cells. Luciferase activity was measured at the indicated time points post-electroporation. Raw luciferase activity of individual SLA variants, including (**A**) SLA^{Anc}, (**B**) SLA^{Int}, (**C**), SLA^{Con}, and (**D**) SLA^{Alt} is shown. Error bars represent the SD, and the limit of detection is indicated via a dashed line. Data shown represents two independent biological replicates. *$p < 0.05$, **$p < 0.01$, ***$p < 0.001$, ****$p < 0.0001$.

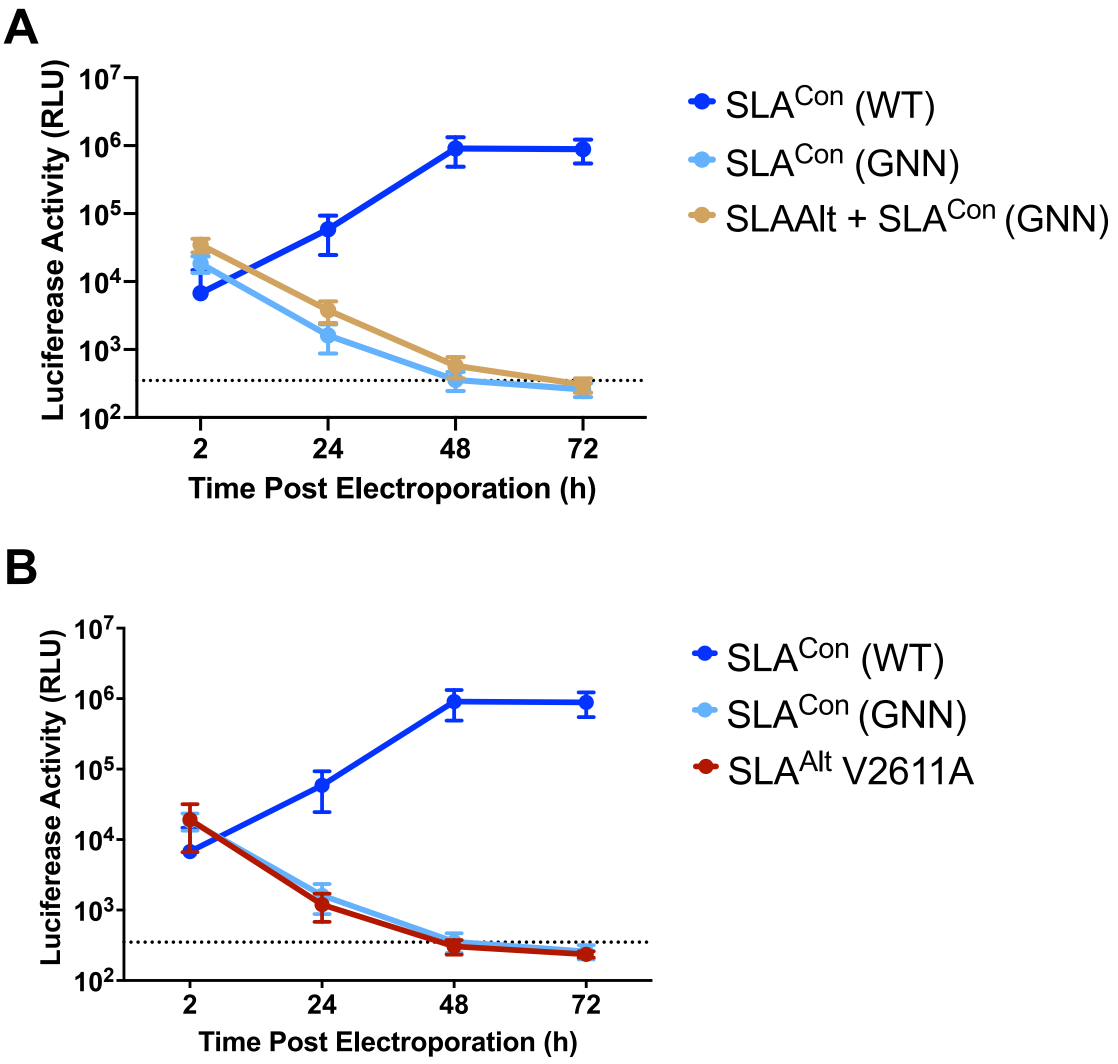

Figure S4. *Trans*-complementation or introduction of the V2611A polymorphism does not rescue SLA^{Alt} subgenomic replicon RNA accumulation cell culture. A549 cells were electroporated with replication-competent (WT) or replication-defective (GNN) SLA^{Con} replicon as controls. SLA^{Alt} rescue was attempted by either (**A**) complementation of the SLA^{Alt}-encoding subgenomic replicon with a GNN SLA^{Con} replicon in *trans*, or (**B**) introduction of the V2611A amino acid mutation into the SLA^{Alt}-encoding subgenomic replicon. Luciferase activity was measured at the indicated time points post-electroporation. All data are representative of at least 2 independent biological replicates and error bars represent the standard error of the mean (SD). The limit of detection is indicated via the dashed line.

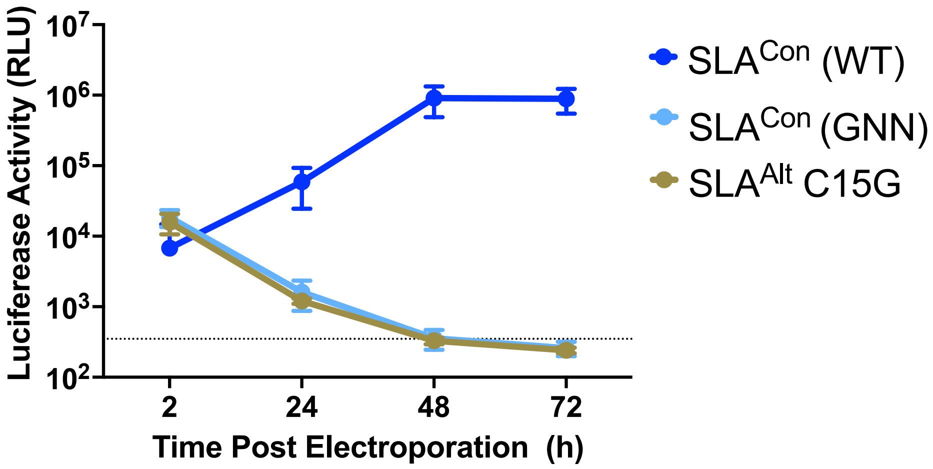

Figure S5. The SLA^{Alt} C15G subgenomic replicon is not replication-competent. Replication-competent (WT) or replication-defective (GNN) SLA^{Con} and a SLA^{Alt} C15G subgenomic replicon RNA were electroporated into A549 cells. Luciferase activity was measured at the indicated time points post-electroporation. All data are representative of a least 2 independent biological replicates and error bars represent the SD. The limit of detection is indicated via a dashed line.

Supplementary Tables

Table S1. Primers used in cloning.

Primer	Sequence (5′-3′)
SLA-G19A-C41U-RPCR-Fwd	CAGTTCGAGTCTGAAGCGAAAGCTAGCAAC
SLA-G19A-C41U-RPCR-Rev	TCGCAGTCTGACTCACACAGATCAACAACTC
SLA-G50A-A56G-RPCR-Fwd	TAACAACAGTATCAACAGGTTTTATTTTG
SLA-G50A-A56G-RPCR-Rev	GCTCTCGCTTCAGACTCGAACTG
SLA-Alt-RPCR-Fwd-1	GCTGACTCAGACTGCGACAGTTCG
SLA-Alt-RPCR-Rev-1	AACAGTAACAACTCTATAGTGTCCCC
pos363IVT-SLAalt-RPCR-For	GTTGCTGACTCAGACTGCGACAGTTCG
pos363IVT-SLAalt-RPCR-Rev	AGTAACAACTCTATAGTGAGTCGTATTAGAATTCG
ZIKV-5(+)-GA-Insert-Fwd	TACGACTCACTATAG AGTTGTTGATCTGTGTGAATCAG
ZIKV-5(+)-GA-Insert-Rev	ATGCCATGCCGACCC CAGCATGGCAGCCAG;
pUC18-SVP-RPCR-Fwd	GGGTCGGCATGGCATCTC
(+)163nt Vector RPCR Rev	CTATAGTGAGTCGTATTAGAATTCGTAATCATGG
ZIKV-WT-IVT-T7II-For	GACTCACTATTAGTTGTTGATCTG
ZIKV-Alt-IVT-T7II-For	GACTCACTATTAGTTGTTACTGTTG
ZIKV-puc18-IVT-T7II-Rev	GTATTAGAATTCGTAATCATGG

Table S2. Amino Acid Polymorphisms in SLA[Alt] strains compared to ZIKV[PR] (SLA[Con])

	Capsid			prM	E										NS1									NS2A	NS3						NS4B					NS5									
	76	80	107	273	313	323	442	503	520	613	620	623	739	794	795	892	916	970	1005	1050	1095	1107	1118	1263	1622	1857	1867	1938	2123	2167	2295	2313	2317	2419	2445	2594	2611	2807	2809	2833	2842	2974	2975	3162	3334
**Puerto Rico/2015[a] (KX377337)**	**E**	**T**	**D**	**S**	**V**	**V**	**I**	**V**	**D**	**H**	**V**	**A**	**F**	**A**	**D**	**G**	**Y**	**S**	**R**	**T**	**T**	**C**	**R**	**A**	**D**	**H**	**S**	**D**	**F**	**L**	**I**	**A**	**T**	**D**	**I**	**Y**	**V**	**S**	**H**	**P**	**I**	**N**	**M**	**S**	**V**
Brazil/2015[b] (KX520666)	•	I	•	R	•	•	•	•	•	•	•	•	•	•	•	W	•	•	•	•	•	•	•	V	G	•	•	•	L	M	•	•	•	•	•	H	A	•	•	•	V	•	•	P	•
Brazil/2015 (KU321639)	•	I	•	•	I	•	•	•	•	•	•	•	•	•	•	W	H	•	•	•	•	•	•	•	•	Y	•	•	•	•	M	•	•	•	M	•	A	•	•	•	•	•	•	•	•
Haiti/2014 (KU509998)	•	I	•	•	•	•	•	•	•	•	•	•	•	•	•	W	H	•	•	•	•	•	•	•	•	Y	•	•	•	•	M	•	•	•	M	•	A	•	•	•	•	•	•	•	•
Venezuela/2016 (KU744693)	D	I	•	•	•	A	L	A	A	H	G	G	I	G	G	W	•	W	Q	A	•	S	Q	•	•	Y	R	G	•	•	M	P	S	E	M	•	A	A	K	A	•	I	T	•	A
Haiti/2016 (KX051563)	•	I	•	•	•	•	•	•	•	•	•	•	•	•	•	W	H	•	•	•	•	•	•	•	•	Y	•	•	•	•	M	•	•	•	•	•	A	•	•	•	•	•	•	•	•
Colombia/2016 (KX247646)	•	I	E	•	•	•	•	•	•	•	•	•	•	•	•	W	•	•	•	•	•	•	W	•	•	•	•	•	•	•	•	•	•	•	•	•	A	•	•	•	•	•	•	•	•
Venezuela/2016 (KX702400)	D	I	E	•	•	•	•	•	•	•	•	•	•	•	•	W	•	•	•	•	P	•	W	•	•	•	•	•	•	•	•	•	•	•	•	•	A	•	•	•	•	•	•	•	•
Venezuela/2015 (KX893855)	X	I	E	•	•	•	•	•	•	•	•	•	•	•	•	W	•	•	•	•	P	•	W	•	•	•	•	•	•	•	•	•	•	•	•	•	A	•	•	•	•	•	•	•	•
Brazil/2016 (MT483911)	E	I	•	•	•	•	•	•	•	•	•	•	•	•	•	W	•	•	•	•	•	•	•	•	•	•	•	•	•	•	•	•	•	•	M	•	A	•	•	•	•	•	•	•	•

[a]ZIKV[PR], [b]ZIKV[BR]
